# Identification of GA-Binding Protein Transcription Factor Alpha Subunit (GABPA) as a Novel Bookmarking Factor

**DOI:** 10.3390/ijms20051093

**Published:** 2019-03-04

**Authors:** Shunya Goto, Masashi Takahashi, Narumi Yasutsune, Sumiki Inayama, Dai Kato, Masashi Fukuoka, Shu-ichiro Kashiwaba, Yasufumi Murakami

**Affiliations:** 1 Department of Biological Science and Technology, Faculty of Industrial Science and Technology, Tokyo University of Science, 6-3-1 Niijuku, Katsushika-ku, Tokyo 125-8585, Japan; 8315702@ed.tus.ac.jp (S.G.); 8316630@ed.tus.ac.jp (M.T.); 8317664@ed.tus.ac.jp (N.Y.); 8318510@ed.tus.ac.jp (S.I.); kashiwaba@rs.tus.ac.jp (S.K.); 2 Order-MadeMedical Research Inc., 208Todai-Kashiwa VP, 5-4-19 Kashiwanoha, Kashiwa-shi, Chiba-ken 277-0882, Japan; daikato@omr.co.jp; 3 Department of Molecular Pharmacology, National Institute of Neuroscience, National Center of Neurology and Psychiatry, Tokyo 187-8551, Japan; fukuokamasa@ncnp.go.jp

**Keywords:** mitotic bookmarking, bookmarking factor, histone acetylations, GABPA, SP1, early G1 genes

## Abstract

Mitotic bookmarking constitutes a mechanism for transmitting transcriptional patterns through cell division. Bookmarking factors, comprising a subset of transcription factors (TFs), and multiple histone modifications retained in mitotic chromatin facilitate reactivation of transcription in the early G1 phase. However, the specific TFs that act as bookmarking factors remain largely unknown. Previously, we identified the “early G1 genes” and screened TFs that were predicted to bind to the upstream region of these genes, then identified GA-binding protein transcription factor alpha subunit (GABPA) and Sp1 transcription factor (SP1) as candidate bookmarking factors. Here we show that GABPA and multiple histone acetylation marks such as H3K9/14AC, H3K27AC, and H4K5AC are maintained at specific genomic sites in mitosis. During the M/G1 transition, the levels of these histone acetylations at the upstream regions of genes bound by GABPA in mitosis are decreased. Upon depletion of GABPA, levels of histone acetylation, especially H4K5AC, at several gene regions are increased, along with transcriptional induction at 1 h after release. Therefore, we proposed that GABPA cooperates with the states of histone acetylation to act as a novel bookmarking factor which, may negatively regulate transcription during the early G1 phase.

## 1. Introduction

Cell-type-, tissue-, and developmental stage-specific gene expression patterns are tightly controlled by transcriptional regulatory machinery consisting of specific transcription factors (TFs), epigenetic modifications, and higher-order chromatin structures. However, these cellular environments markedly change accompanying the breakdown of the nuclear envelope and chromatin condensation in mitosis, and are then restored upon chromatin decondensation and the reorganization of chromatin structures during the M/G1 transition [1].

An early study showed that when cells enter mitosis, transcription is temporally silenced by the displacement of RNA polymerases and TFs from chromatin [2]. When cells exit from mitosis, transcription is faithfully restarted by reorganization of the transcriptional regulatory machineries on the chromatin of daughter cells [3]. These events may play an important role in the faithful transmission of parental cell function to the daughter cells after cell division. Although the mechanisms by which cells reactivate the specific transcriptional patterns have not been fully elucidated, a process termed “mitotic bookmarking” has been proposed to be involved in the faithful transmission of transcriptional patterns across cell division [4]. In particular, mitotic bookmarking comprises the mechanisms by which histone modifications and transcriptional regulatory factors (bookmarking factors) are retained in specific or nonspecific genomic sites throughout mitosis, facilitating immediate transcriptional reactivation after cell division [4,5,6,7,8,9,10,11,12,13,14,15,16,17,18,19,20]. In addition, recently developed highly sensitive assays showed that the transcription profile is largely retained at a low level during mitosis [21]. Other studies, with respect to transcription during the early G1 phase, indicated the existence of hierarchies of reactivation timing in individual genes along with different reactivation patterns during the M/G1 transition [21,22]. However, the interrelationships among these insights and the transmission of transcriptional memory throughout cell division are not yet well understood.

Previously we identified a variety of genes preferentially reactivated at the early G1 phase (early G1 genes) following establishment of a method for comprehensive gene expression analysis using nascent mRNAs specifically isolated from living mammalian cells and obtaining detailed expression profiles during the M/G1 transition. Furthermore, we discovered the motifs that were predicted to be bound by GA-binding protein transcription factor alpha subunit (GABPA) and Sp1 transcription factor (SP1) at upstream regions of all early G1 genes analyzed [23].

GABPA plays an important role in regulating genes involved in various biological processes such as embryonic development, cell differentiation, cell cycle, and mitochondrial biogenesis [24,25,26,27,28]. SP1, one of the best-studied TFs, regulates numerous genes involving broad biological processes [29]. During the M/G1 transition, SP1 is phosphorylated by cyclin-dependent kinase 1 (CDK1) to dissociate from chromatin and is dephosphorylated by type 2A serine/threonine protein phosphatase (PP2A) to rapidly rebind DNA at the early G1 phase [30]. In addition, although regulation mechanisms have been reported whereby a number of nuclear factors including SP1 are actively evicted from chromatin by mitosis-specific phosphorylation, the possibility exists that a portion of these factors may actively or stochastically maintain the default states by being protected from such phosphorylation [31,32,33,34,35,36,37,38]. Therefore, default states of these factors may bind to mitotic chromatin. Furthermore, GABPA directly interacts with SP1 on the utrophin promoter and activates the transcription of utrophin [39]. GABPA and SP1 have also been reported to synergistically facilitate the transcription of the thymidylate synthase (TS) gene by binding to its promoter [40].

Thus, we hypothesized that GABPA cooperates with SP1 in regulating early G1 gene transcription as a bookmarking factor. In this study, we investigated whether GABPA and SP1 are involved in mitotic bookmarking. Moreover, we also investigated whether these factors regulate the genes reactivated during the M/G1 transition by cooperating with multiple histone acetylation marks that have been reported to be involved in mitotic bookmarking. First, we showed that GABPA, but not SP1, binds to upstream regions of the several genes reactivated during the M/G1 transition in mitotic-arrested cells. Second, the levels of multiple histone acetylations such as H3K9/14AC, H3K27AC, and H4K5AC at the upstream regions of the genes bookmarked by GABPA are higher than those unbookmarked by GABPA in mitotic-arrested cells. Notably, we also found that the levels of multiple histone acetylations at the genes bookmarked by GABPA are decreased during the M/G1 transition, which is inconsistent with previous studies wherein histone acetylation levels were elevated during the M/G1 transition. Finally, we indicated that *Gabpa* knockdown affects histone acetylation at upstream regions of the studied genes and upregulates transcriptional reactivation during the M/G1 transition. Our results suggest that GABPA functions as a novel bookmarking factor that marks the various genes reactivated during the M/G1 transition along with multiple histone acetylations. Moreover, we propose that GABPA may regulate transcriptional reactivation of these genes by controlling histone acetylation states.

## 2. Results

### 2.1. A Fraction of GABPA and SP1 Binds to Mitotic Chromatin

**Figure 1:**
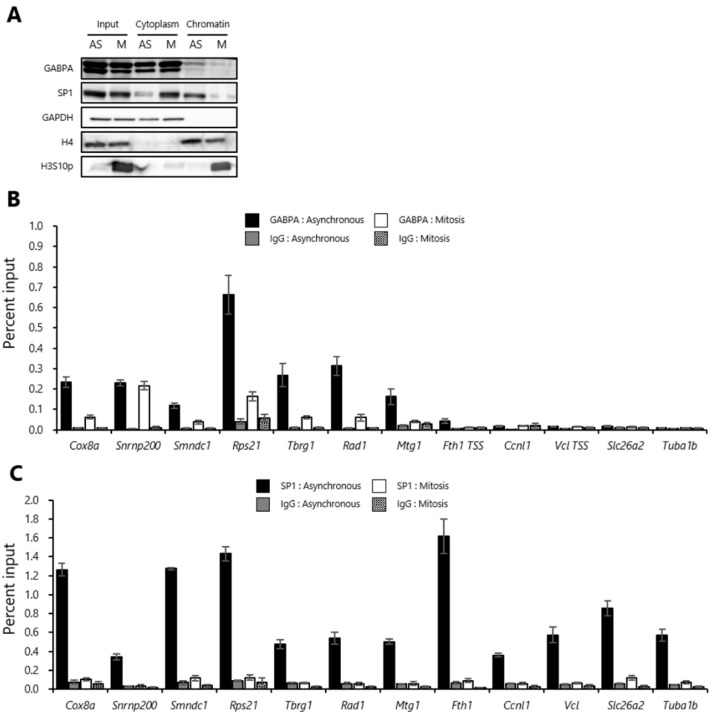
GA-binding protein transcription factor alpha subunit (GABPA), but not Sp1 transcription factor (SP1), partly binds to the analyzed genes in mitosis. (**A**) Protein levels of GABPA and SP1 in the cytoplasmic and chromatin fractions. Asynchronous (AS) and mitotic-arrested (M) tsFT210 cells were fractionated into cytoplasmic and chromatin fractions. The fractions were then analyzed by western blotting with the indicated antibodies. Glyceraldehyde-3-phosphate dehydrogenase (GAPDH) and histone H4 (H4) were used as controls for the cytoplasmic and chromatin fractions, respectively. Phosphorylation at Ser10 of histone H3 (H3S10p) is a marker of mitotic cells. (**B**,**C**) Bindings of GABPA and SP1 at the upstream regions of the genes reactivated during the M/G1 transition in asynchronous and mitotic-arrested tsFT210 cells. Asynchronous and mitotic-arrested tsFT210 cells were subjected to chromatin immunoprecipitation using antibodies against GABPA and SP1, followed by quantitative PCR using primer sets specific for the upstream regions of the several genes reactivated during the M/G1 transition. Normal rabbit IgG was used as a negative control. Error bars denote SEM (*n* = 3).

To assess whether GABPA and SP1 have the potential to act as bookmarking factors, we investigated their binding to mitotic chromatin. For this purpose, it was necessary for cells to be highly arrested in mitosis. Previously, we have demonstrated a method to synchronize the cell cycle in mitosis by a combination of temperature-sensitive CDC2 inactivation and nocodazole—an inhibitor of microtubule polymerization [23]. We prepared asynchronous and mitotic-arrested tsFT210 cells using this method. To evaluate the purity of the mitotic population, first, we performed immunostaing with antiphosphorylation at Ser10 of histone H3 (H3S10p) antibody and counterstaining of DNA with DAPI using asynchronous tsFT210 cells. We showed that the average percentage of mitotic cells in asynchronous tsFT210 cells is approximately 12% (Supplementary Figure S1). Second, we also performed fluorescence-activated cell sorting (FACS) analysis using propidium iodide and anti H3S10p antibody using gating criteria suitable for the result from supplemental Figure S1. We confirmed that the percentage of the mitotic cells in the cells synchronized by this method is approximately 80% (Supplementary Figure S2). Then, we isolated cytoplasmic and chromatin fractions from these cells and analyzed the protein levels of GABPA and SP1 in each fraction by Western blot analysis. In asynchronous cells, a portion of total GABPA protein was detected in the chromatin fraction, whereas a large part was located in the cytoplasmic fraction (Figure 1A). Similarly, we also observed a portion of total GABPA protein in the chromatin fraction derived from mitotic-arrested tsFT210 cells (Figure 1A). Compared to GABPA, SP1 was detected predominantly in the chromatin fraction in asynchronous cells. Although the majority of SP1 protein was detected in the cytoplasmic fraction, a portion still remained in the chromatin fraction in mitotic-arrested tsFT210 cells (Figure 1A).

### 2.2. GABPA, but Not SP1, Binds to Upstream Regions of the Various Genes Reactivated during the M/G1 Transition in Mitotic-Arrested tsFT210 Cells

Next, we examined whether GABPA and SP1 bind to upstream regions of genes that are reactivated during the M/G1 transition. Then, we performed a chromatin immunoprecipitation (ChIP) assay coupled with quantitative PCR using asynchronous and mitotic-arrested tsFT210 cells. Several genes reactivated during the M/G1 transition were selected from our previous study of genome-wide gene expression analysis by DNA microarray using nascent mRNA [23], and binding of GABPA and SP1 proteins to their upstream regions was examined using primer sets specific for the regions near the transcription start site. In asynchronous cells—taking into account that both the absolute percent input values and relative enrichment values exceeded two-fold versus IgG—the upstream regions of various tested genes were robustly bound by GABPA (Figure 1B, Supplementary Figure S3A left side). Furthermore, as judged in the same classification of the asynchronous cells, the upstream regions of selected genes which exhibited GABPA binding in asynchronous cells also showed GABPA bindings in mitotic-arrested tsFT210 cells (Figure 1B, Supplementary Figure S3A right side). In comparison, marked SP1 binding was detected only in asynchronous cells in these regions except for *Fth1* and *Slc26a2* genes (Figure 1C, Supplementary Figure S3B). These results suggested that GABPA, but not SP1, potentially functions as a bookmarking factor for the various genes reactivated during the M/G1 transition. Hereafter, the genes reactivated during the M/G1 transition bound by GABPA in mitosis, those not bound by GABPA in mitosis, and those not bound by GABPA in both asynchronous and mitosis are respectively termed “bookmarked”, “unbookmarked”, and “GABPA-unbound” genes.

### 2.3. Histones at the Sites Bookmarked by GABPA are Highly Acetylated in Mitosis

**Figure 2:**
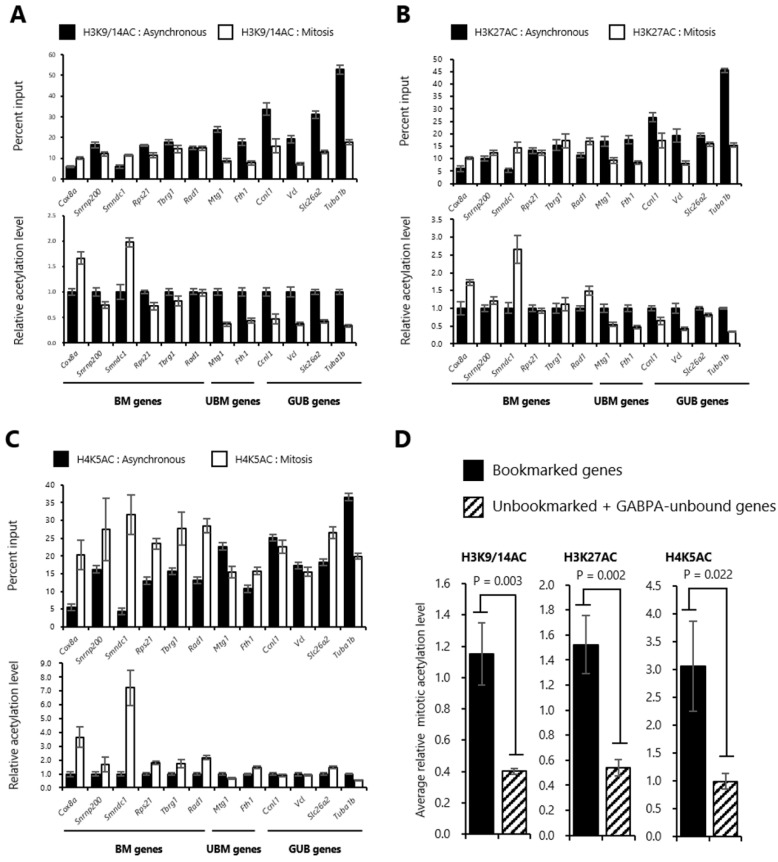
Histones at the sites bookmarked by GABPA are highly acetylated in mitosis. (**A**–**C**) The histone acetylation levels at upstream regions of the genes reactivated during the M/G1 transition in asynchronous and mitotic-arrested tsFT210 cells. Asynchronous and mitotic-arrested tsFT210 cells were subjected to chromatin immunoprecipitation assay using antibodies against H3K9/14AC (**A**), H3K27AC (**B**), and H4K5AC (**C**), followed by quantitative PCR using primer sets specific for the upstream regions of the bookmarked (BM), unbookmarked (UBM), and GABPA-unbound (GUB) genes. In each figure, the upper graph shows raw data plotted as percent input DNA and the lower graph shows the same data replotted relative to the asynchronous acetylation level. Error bars denote SEM (*n* = 3). (**D**) Comparison of the average relative level of each mitotic histone acetylation at upstream regions of the bookmarked genes with those of unbookmarked and GABPA-unbound genes. Error bars denote SEM (*n* = 3). Statistical significance was analyzed using a one-tailed Student’s *t*-test.

It was previously shown that transcriptionally active gene regions are marked by histone acetylation during interphase, whereas in cells entering mitosis these histone acetylation sites are globally hypoacetylated [41]. However, several transcriptional regulatory regions such as enhancers and promoters retain a higher level of histone acetylation in mitosis than other genomic regions [42]. Furthermore, as these regions partially overlapped with the sites bound by bookmarking factors in mitosis, it was considered that the binding of bookmarking factors is related to histone acetylation states in mitotic chromatin [7,8,20]. Thus, we compared histone acetylation levels at upstream regions of the bookmarked genes with those of unbookmarked and GABPA-unbound genes in mitotic-arrested tsFT210 cells. As acetylation of histones H3K9/14, H3K27, and H4K5 is thought to be involved in mitotic bookmarking [7,8,20,22], we examined levels of these acetylated histones in asynchronous and mitotic-arrested tsFT210 cells. First, we performed Western blotting with antibodies against H3K9/14AC, H3K27AC, and H4K5AC using the same samples as in Figure 1A. Consistent with previous studies, we found that the levels of these histone acetylation marks are decreased in mitotic-arrested tsFT210 cells compared with those in asynchronous cells (Supplementary Figure S4). Next, to examine the levels of these histone acetylations at upstream regions of the genes reactivated during the M/G1 transition in asynchronous and mitotic-arrested tsFT210 cells, we performed ChIP assay coupled with quantitative PCR using the same primer sets as in Figure 1B. We observed that H3K9/14AC and H3K27AC levels at the upstream regions of the bookmarked genes in mitotic arrested tsFT210 cells were comparable (or higher) to those in asynchronous cells (Figure 2A,B, left side). In contrast, the levels of these histone acetylation marks at the unbookmarked and GABPA-unbound genes in mitotic-arrested tsFT210 cells were mostly low compared with those in asynchronous cells (Figure 2A,B, right side). Similarly, H4K5AC levels at the bookmarked genes were markedly higher in mitotic-arrested tsFT210 cells compared with those in asynchronous cells (Figure 2C, left side), whereas the acetylation levels at the upstream regions of the unbookmarked and GABPA-unbound genes were comparable between mitotic-arrested tsFT210 cells and asynchronous cells (Figure 2C, right side). To evaluate the relationship between the presence of GABPA in mitosis and these histone acetylations, we calculated the average level of each histone acetylation in mitotic-arrested tsFT210 cells relative to that in asynchronous cells and compared between the presence and absence of GABPA binding in mitosis. We found that these histone acetylation levels were all significantly higher (approximately two- to three-fold) at the bookmarked genes rather than the unbookmarked and GABPA-unbound genes (Figure 2D). To exclude the possibility that the changes in histone acetylation status at the upstream regions of the studied genes were caused by alteration of nucleosome occupancy, we performed ChIP assays using an anti-histone H3 antibody coupled with quantitative PCR using the same primer sets as in Figure 1B. Our results revealed no significant difference of the level of H3 between the bookmarked genes, unbookmarked, and GABPA-unbound genes (Supplementary Figure S5). Therefore, in this study, the contribution of differential nucleosome occupancy affecting the histone acetylation levels at the studied genes was considered to be negligible. These results suggested that GABPA binding at high retention with multiple histone acetylation sites in mitosis may comprise novel mitotic marks that potentially function as mitotic bookmarking at the upstream region of genes reactivated during the M/G1 transition.

### 2.4. Histones at the Sites Bookmarked by GABPA are Deacetylated during the M/G1 Transition

**Figure 3:**
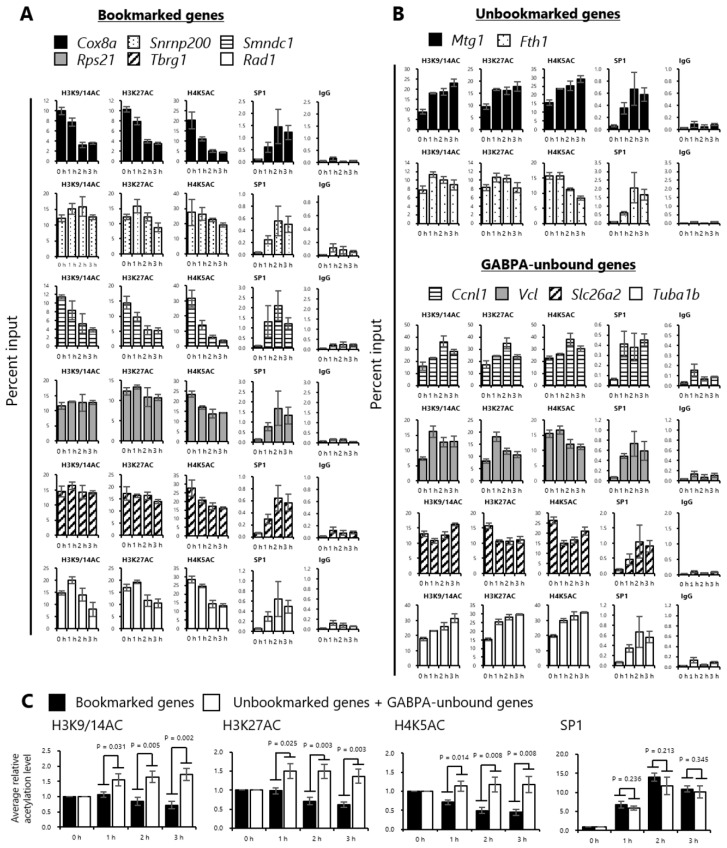
Histones at the sites bookmarked by GABPA are deacetylated during the M/G1 transition. (**A**,**B**) Histone acetylation and SP1 binding in cells at indicated time points after release from mitotic synchronization. Mitotic-arrested tsFT210 cells were released and harvested at indicated time points, then subjected to chromatin immunoprecipitation assay using antibodies against H3K9/14AC, H3K27AC, H4K5AC, and SP1, followed by quantitative PCR using primer sets specific for upstream regions of the bookmarked (**A**), unbookmarked (**B**), and GABPA-unbound genes. Normal rabbit IgG was used as a negative control. The data of 0 h are the same as those of Figure 1 and Figure 2. Error bars denote SEM (*n* = 3). (**C**) Comparison of the average relative level of each histone acetylation and SP1 binding in cells at indicated time points after release from mitotic synchronization at the bookmarked gene regions vs. those unbookmarked and GABPA-unbound gene regions. Error bars denote SEM (*n* = 3). Statistical significance was analyzed using a one-tailed Student’s *t*-test.

Previous studies showed that a known bookmarking factor, bromodomain protein 4 (BRD4), binds to the transcriptional start sites of several “M/G1 genes” in mitosis, then BRD4 binding on these regions increases coinciding with the increase of histone H3/H4 acetylation during the M/G1 transition, thereby recruiting positive transcription elongation factor b (P-TEFb) and reactivating the transcription of “M/G1 genes” [7,8]. Based on our findings that the bookmarked gene regions have high histone acetylation levels in mitosis compared with those of unbookmarked and GABPA-unbound gene regions, and that SP1 bound to the majority of the tested genes in asynchronous cells but not in mitotic-arrested cells, we hypothesized that the levels of histone acetylation and SP1 binding are elevated immediately during the M/G1 transition preferentially at the bookmarked gene regions, such as genes bookmarked by BRD4. Consequently, these genes rapidly reactivate their transcription. To test this hypothesis, we synchronized cells in mitosis as described in Figure 1, then released them by nocodazole wash out and harvested the cells 1, 2, and 3 h after release. The cells were then subjected to ChIP assay using antibodies against H3K9/14AC, H3K27AC, H4K5AC, and SP1, followed by quantitative PCR using primer sets specific for the upstream regions of the bookmarked, unbookmarked, and GABPA-unbound genes. Notably, the H3K9/14, K27, and H4K5 acetylation levels at the upstream region of the bookmarked genes were retained or decreased after release from mitotic arrest (Figure 3A). In contrast, the acetylation levels at the unbookmarked and GABPA-unbound genes were increased, retained, or gradually decreased through G1 phase progression (Figure 3B). Alternatively, the binding of SP1 was increased at 1 h after release from mitotic arrest at the upstream regions of all tested genes (Figure 3A,B). To evaluate the relationship among bookmarking by GABPA, histone acetylations, and SP1 recruitment, we calculated the average level of each histone acetylation and SP1 binding, and compared these levels between the bookmarked genes, unbookmarked, and GABPA-unbound genes. We found that the levels of all histone acetylation marks at 1 to 3 h after release were significantly lower at the bookmarked genes than at the unbookmarked and GABPA-unbound genes (Figure 3C). However, there was no significant difference in SP1 binding (Figure 3C). These results suggested that GABPA binding in mitosis may be involved in the suppression of multiple histone acetylation marks at the upstream region of a portion of the early G1 genes during the M/G1 transition, whereas SP1 binding is independent of GABPA binding in mitosis.

### 2.5. Gabpa Knockdown Increases Histone Acetylation Levels at Several Genes Reactivated during the M/G1 Transition

**Figure 4:**
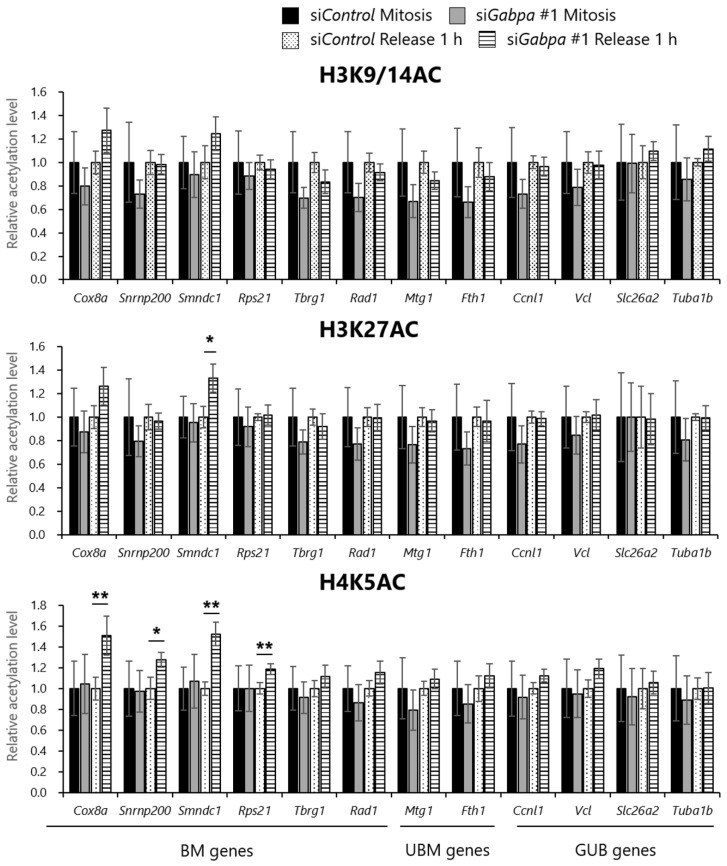
*Gabpa* knockdown initially increases H4K5 acetylation level at bookmarked genes in early G1 phase. Mitotic-arrested tsFT210 cells, and those 1h after release from mitotic arrest, were harvested, then subjected to chromatin immunoprecipitation assay using antibodies against to H3K9/14AC, H3K27AC, and H4K5AC, followed by quantitative PCR using primer sets specific for upstream regions of the bookmarked (BM), unbookmarked (UBM), and GABPA-unbound (GUB) genes. Error bars denote SEM (*n* = 3) asterisks indicate significance using a one-tailed Student’s *t*-test: (**) *p* < 0.05, (*) *p* < 0.1.

Unlike previous studies, our data indicated that the levels of H3K9/14AC, H3K27AC, and H4K5AC at the upstream regions of the bookmarked genes decreased during the M/G1 transition. To determine whether GABPA is involved in deacetylation of histones H3 and H4 during the M/G1 transition, we investigated the effect of *Gabpa* knockdown on histone acetylation states. First, we confirmed the downregulation efficiency of siRNA against *Gabpa*. We downregulated GABPA in tsFT210 cells by siRNA and synchronized them in mitosis. Cells were harvested 1 h after release from mitotic arrest and then subjected to further assays. As shown in Supplementary Figure S6A, the protein levels of GABPA detected by Western blotting were efficiently decreased in cells transfected with siRNA against *Gabpa*. Second, to examine the effect of *Gabpa* knockdown on the acetylation levels of histone H3K9, H3K27, and H4K5 during the M/G1 transition, we performed a ChIP assay coupled with quantitative PCR using the same primer sets as in Figure 1B. As shown in Figure 4 and supplementary Figure S7, we detected significant increase (*p* < 0.05) of H4K5AC at *Cox8a*, *Smndc1*, and *Rps21* (bookmarked genes) in the *Gabpa* knockdown cells at 1 h after release. Similar trends were observed for H3K27AC at *Smndc1* and H4K5AC at *Snrnp200*, although they did not reach significance (*p* < 0.1). However, we could not observe an effect of *Gabpa* knockdown on the levels of these histone acetylation marks in mitosis, or those of H3K9/14AC at 1 h after release. In contrast, in asynchronous *Gabpa* knockdown cells, we detected an increase of these histone acetylation levels at *Cox8a* and an increase of H3K9/14AC or H3K27AC at several bookmarked, unbookmarked, or GABPA-unbound genes (Supplementary Figure S8). Similar trends were obtained with the same assay using a different siRNA against *Gabpa* (Supplementary Figures S9–S11). To exclude the possibility that the changes in histone acetylation status caused by *Gabpa* knockdown were due to altered cell cycle progression, we compared the cell cycle profile between cells transfected with siRNA against *Gabpa* and negative control siRNA by flow cytometry. We found that there were no significant differences in asynchronous, mitotic-arrested tsFT210 cells and in cells 1 h after release from mitotic arrest (Supplementary Figure S6B). These results suggested that the histone acetylation levels at the several upstream regions of the studied genes are coordinated by GABPA, especially histone H4K5 acetylation levels at those of various bookmarked genes are preferentially affected by GABPA during the M/G1 transition.

### 2.6. Gabpa Knockdown Increases Transcriptional Induction in the Early G1 Phase

**Figure 5:**
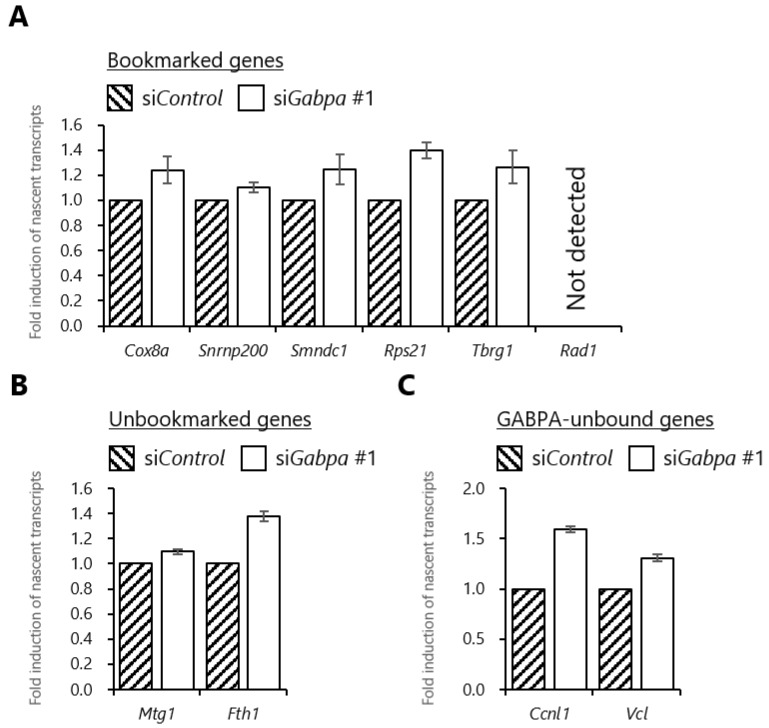
*Gabpa* knockdown increases transcriptional induction at the early G1 phase. (**A**,**B**) Relative nascent transcriptional induction after release from mitotic synchronization in *Gabpa* knockdown cells. tsFT210 cells transfected with siRNA against *Gabpa* or negative control siRNA were arrested in mitosis, then released and harvested 1 h after release. Total cellular RNAs were then subjected to quantitative RT-PCR using primer sets specific for the nascent transcripts of the bookmarked (**A**), the unbookmarked (**B**), and GABPA-unbound (**C**) genes. Data represent relative values of 1 h versus 0 h and normalized to negative control siRNA. Error bars denote SEM (*n* = 3).

Generally, mitotic bookmarking factors are considered to bind to target genes in mitosis and facilitate their transcriptional reactivation after mitosis. However, previous reports suggested that runt-related transcription factor 2 (RUNX2) acts as a transcriptionally suppressive bookmarking factor [43,44,45]. Notably, GATA transcription factor 1 (GATA1) was suggested to be a unique bookmarking factor functioning as both activator and suppressor [10]. In contrast, in general, histone deacetylation is associated with transcriptional suppression, and our results indicated that GABPA binding in mitosis is involved in histone deacetylation during the M/G1 transition. Thus, we conjectured that GABPA functions as a transcriptional suppressor during the M/G1 transition by regulating the deacetylation of histone H3 and H4. Accordingly, we examined the effect of *Gabpa* knockdown on transcriptional reactivation of the genes bookmarked, unbookmarked or unbound by GABPA during the M/G1 transition by quantitative reverse transcription (RT)-PCR analysis. In this analysis, we used primers specific for pre-mRNAs to prevent detection of mature mRNAs that may have been carried over from a prior G2 phase. Prior to the analysis, we validated the expression profiles of the studied genes using primers specific for pre-mRNAs. Our result shows that the expression profiles of those genes were consistent with our previous microarray analysis of nascent mRNA [23] (Supplementary Figure S12). Subsequently, knockdown of *Gabpa* resulted in the upregulation of pre-mRNA levels of the bookmarked genes at 1 h after release (Figure 5A) in addition to those of the unbookmarked and GABPA-unbound genes (Figure 5B,C). These results suggested that at 1 h after release from mitotic arrest, general levels of GABPA may be directly and indirectly involved in transcriptional suppression of the various tested genes. Whereas, these results raised the possibility that increasing of H4K5 acetylation states at the upstream regions of the various bookmarked genes at 1 h after release by knockdown of *Gabpa* may not affect transcriptional reactivation at this time point.

## 3. Discussion

From our previous screening of early G1 genes using nascent mRNA [23], GABPA was predicted to be a candidate novel bookmarking factor that may bind to the upstream region of most of the early G1 genes. In the present study, we experimentally identified GABPA as a novel bookmarking factor, which indicated that our approach is suitable for identifying novel bookmarking factors. Analysis of a variety of cell lines by this method may therefore lead to the identification of novel common or cell type-specific bookmarking factors.

Our ChIP analysis showed that GABPA preferentially bound to a subset of genes reactivated during the M/G1 transition. However, the genes bound by GABPA in mitosis are not fully identified and the mechanisms by which GABPA binds to limited regions in mitosis are unknown. In comparison, previous studies have revealed the genome-wide distributions of other bookmarking factors in mitosis. Hematopoietic transcription factor GATA1 tends to bind to genes of nuclear factors essential for erythroid differentiation in interphase and mitosis cells although there is no marked difference in chromatin features between the binding sites in “interphase cells only” (genes unbookmarked by GATA1) and those in “interphase and mitosis cells” (genes bookmarked by GATA1) [10]. Similarly, the pioneer factor FoxA1 bookmarks the genes important for liver differentiation, albeit no marked difference in chromatin features has been detected between the genes unbookmarked and those bookmarked by FoxA1 [12]. Recently, several genome-wide studies indicated that stem cell regulators, such as SOX2, OCT4, and KLF4, bookmark stem cell-related genes, although these findings remain controversial [18,20,46]. Estrogen related receptor, beta (ESRRB) also bookmarks stem cell-related genes; moreover, the regions bookmarked by ESRRB contain extra bases of the consensus sequence present at the 5′ end of the canonical ESRRB binding motif [19]. To obtain deeper insight regarding GABPA as a bookmarking factor, genome-wide analysis by ChIP-Seq of GABPA distribution is required to reveal which biological processes are regulated by the genes bookmarked by GABPA, and whether specific features of the DNA sequence are required for bookmarking by GABPA.

We also revealed that multiple histone acetylation marks are retained or increased at upstream regions of the bookmarked genes in mitotic-arrested tsFT210 cells relative to those in asynchronous cells, whereas global histone acetylation levels are decreased during mitosis. The correlation between GABPA binding and histone acetylation states in asynchronous cells has been reported [47], whereas those in mitotic cells are not well understood. Thus, further genome-wide studies of the relationship between GABPA and multiple histone acetylations may reveal the novel transcriptional mechanisms regulated by GABPA. Moreover, the relationship between bookmarking factors and histone modification has also been studied previously. In particular, BRD4 recruitment at mitotic chromatin is mediated by histone acetylation and BRD4 binding on M/G1 genes facilitates postmitotic transcriptional reactivation [7,8]. Histone acetyl transferase p300 contributes to form preinitiation complexes (PICs) containing RNA polymerase II, TBP, and acetylated H3/4 in mitosis at specific genes, with the formed PICs in turn facilitating postmitotic transcriptional reactivation [15]. However, we did not observe preferential binding of BRD4 and RNA polymerase II at upstream regions of the bookmarked genes in the present study (Supplementary Figures S13 and S14). Thus, our findings supported the possibility that the presence of a novel mitotic bookmarking mechanism by GABPA cooperates with multiple histone acetylations independent of BRD4 and RNA polymerase II retention at mitotic chromatin.

Previous studies indicated that histone acetylations at several upstream regions of genes is elevated during the M/G1 transition [7,8]. Conversely, we observed that the levels of multiple histone acetylations at the upstream region of the bookmarked genes were decreased after release from mitotic arrest. The reason for this was not obvious, because although deacetylation of histones is generally considered to decrease transcriptional activity, our data indicated that the bookmarked genes were rapidly reactivated at 1 h after release from mitotic arrest. We consider that a regulatory mechanism may exit involving other regulatory factors, such as Polycomb group (PcG) and Trithorax group (TrxG) proteins, which may explain the observed phenomenon. PcG proteins catalyze suppressive histone modification, and then suppress transcription [48]. In contrast, TrxG proteins catalyze active histone modifications and then activate transcription [49]. Moreover, the opposing activity of factors and histone modifications can antagonize each other, allowing PcG and TrxG to thus dynamically balance transcriptional activity [50]. Notably, these mechanisms are well-conserved in multicellular organisms and thought to be involved in the maintenance of epigenetic memory during mitosis [51]. In interphase cells, it is reported that GABPA indirectly interacts with histone deacetylase 3 (HDAC3) at the *Skp* promoter, with the resultant complex suppressing *Skp* gene transcription [52]. In addition, it is also reported that GABPA regulates acetylcholine receptor (AChR) gene expression by opposing functions that recruit histone acetyl transferase (HAT) p300 or histone deacetylase HDAC1 [53]. Thus, in our case, it is possible that some mechanisms exit that are regulated by the balance between activator and repressor to control gene reactivation during the M/G1 transition

The majority of bookmarking factors are known to contribute to rapid transcriptional reactivation during the M/G1 transition. However, only a limited number of bookmarking factors (such as GATA1 and RUNX2) are reported to negatively regulate transcription [8,33,34,35]. Our knockdown experiment of *Gabpa* suggested that, at the early G1 phase, GABPA initially facilitates deacetylation of histone H4K5 and may function as a transcriptional suppressor for bookmarked genes. Thus, we have identified GABPA as a novel bookmarking factor that coordinates histone acetylation states and may suppress transcription during the M/G1 transition. Moreover, although these histone acetylation states between bookmarked genes, and unbookmarked and GABPA-unbound genes are significantly different at the all analyzed time points, these differences at the late time points tended to be greater than early time point (Figure 3). Therefore, H3K9/14 and H3K27 acetylation levels at bookmarked genes may be significantly affected by knockdown of *Gabpa* in cells at 2 or 3 h after release.

In addition, our knockdown experiment of *Gabpa* indicated that there was no significant difference in transcriptional reactivation between the bookmarked and unbookmarked genes. These results raised the possibility that in present study the suppression of transcriptional reactivation at 1 h after release by knockdown of *Gabpa* is due to function of GABPA through cell cycle rather than the mitosis-specific function. However, described in the previous paragraph, the difference of the histone acetylation levels between bookmarked genes, and unbookmarked and GABPA-unbound genes is more different at the late phase of M/G1 transition. Therefore, it is also possible that the apparent effect of *Gabpa* knockdown involved in transcriptional reactivation of bookmarked genes may be detected at late phase of M/G1 transition. To address the issue that how GABPA functions as a bookmarking factor, there are two points of technical limitations. The first is that as our ChIP analysis targeted only limited regions near the transcription start sites, we cannot exclude the possibility that GABPA binds to other proximal or distal genomic regions and participates in mitotic bookmarking. It is generally considered that TF binding sites at proximal regulatory regions are widely distributed from upstream to downstream of the TSS. Indeed, the sites bound by known bookmarking factors in mitosis present at not only proximal promoter but also gene body and enhancer regions [8,11,18,19]. Thus, GABPA might bind at regions around the TSS or enhancers of the genes that we determined as “unbookmarked” or “GABPA-unbound” genes, and act as a bookmarking factor by regulating histone acetylation states. Genome-wide analysis of the distributions of GABPA binding sites by ChIP-Seq may resolve the question of whether GABPA binding at upstream, downstream, or distal regulatory regions is involved in mitotic bookmarking. The second point is that, because conventional gene knockdown by RNAi downregulates gene expression through the cell cycle, it is difficult to be assess the effect of the loss of GABPA in mitosis. Therefore, we cannot exclude the possibility that the depletion of GABPA in interphase (late G1, S, and G2 phases) affects the protein levels of other bookmarking factors, and indirectly leads to increases or decrease in transcriptional reactivation of the unbookmarked genes. To minimize such a possibility, previous studies have attempted to construct cell lines expressing mitotically unstable bookmarking factors that fused the mitosis-specific degradation domain (MD) of cyclin B1 (amino acids 13–91) [8,17,19]. Therefore, we established a cell line that stably expressed MD-fused GABPA; however, we failed to observe the mitosis-specific degradation of MD-fused GABPA (data not shown). Thus, other methods that suppress GABPA specifically in mitosis will be needed to reveal whether GABPA affects transcriptional reactivation of several genes at early G1 phase in an M/G1 transition-specific manner.

In conclusion, we identified GABPA as a novel bookmarking factor that marks the various tested genes in cooperation with multiple histone acetylations in mitosis and may act as a suppressor for genes reactivated during the M/G1 transition by regulating histone H4K5AC deacetylation. More precise analysis of the action of GABPA will provide further understanding of the molecular mechanism of mitotic bookmarking that regulate the transmission of transcriptional memory through cell division.

## 4. Materials and Methods

### 4.1. Cell Culture and Synchronization of tsFT210 Cells

tsFT210 cells—a Cdc2 temperature-sensitive mutant strain of mouse mammary FM3A cells—were cultured and synchronized as described in our previous study [23].

### 4.2. Immunofluorescence

tsFT210 cells were washed once with PBS (−), cross-linked in 4% paraformaldehyde at room temperature for 10 min. The cells were washed twice in PBS (−) and then fixed on a slide glass (MAS coat; Matsunami Glass Ind., Ltd., Osaka, Japan) by incubated at 37 °C for 40 min. The slides were washed twice with PBS (−), followed by permeabilization with 0.1% Triton X-100 at 4 °C for 5 min. After washes with PBS (−), cells were blocked for 1 h with 5% BSA in PBS (−). Primary antibody (anti-H3S10p [CST, Danvers, MA, USA; #53348, 1:1600]) diluted in 1% BSA in PBS (−) was added and incubated at room temperature for 1 h. After three washes with PBS (−), secondary antibodies (coupled to AlexaFluor 564) and DAPI (Wako Pure Chemical Industries Ltd., Osaka, Japan) diluted in 1% BSA in PBS (−) were added and incubated at room temperature for 1 h. The slides were washed again three times with PBS (−) and mounted with fluorescence mounting medium (Agilent Technologies, Santa Clara, CA, USA). The fluorescence images were obtained using a BZ-X700 microscope (Keyence, Osaka, Japan).

### 4.3. Cell Fractionation

Asynchronous or mitotic-arrested tsFT210 cells were collected and fractionated into cytoplasmic and chromatin fractions using an EzSubcell Extract (ATTO, Tokyo, Japan, WSE-7421) following the manufacturer’s protocol.

### 4.4. Western Blotting

Asynchronous, mitotic-arrested, and released cells were lysed in 1% sodium dodecyl sulfate (SDS) in PBS (−) or fractionated into cytoplasmic and chromatin fractions. The lysates were then separated by SDS polyacrylamide gel electrophoresis, transferred to polyvinylidene fluoride membranes, blocked in 5% skim milk, and then probed with antibodies. Immunoreactive bands were visualized using the ECL western detection reagent (GE Healthcare, Chicago, IL, USA) and LAS-4000 pro image analyzer (GE Healthcare). Primary antibodies were used as follows: anti-GABPA (Santa Cruz Biotechnology, Dallas, TX, USA, sc-22810, [1:500]), anti-SP1 (Santa Cruz Biotechnology, sc-17824, [1:1000]), anti-GAPDH (Santa Cruz Biotechnology, sc-32233, [1:1000]), anti-histone H4 (Abcam, Cambridge, UK; ab10158 [1:1000]), anti-H3S10p (CST, Danvers, MA, USA; #53348 [1:1000]), anti-H3K9/14AC (CST, #9677 [1:1000]), anti-H3K27AC (Abcam, ab4729 [1:1000]), and anti-H4K5AC (Abcam, EP1000Y [1:2000]).

### 4.5. Transfection of siRNA into tsFT210 Cells Via Electroporation

tsFT210 cells (3.0 × 10^6^) were mixed with 200 pmol siRNA against *Gabpa* (Thermo Fisher Scientific, Waltham, MA, USA; Stealth siRNAs MSS274443(#1), MSS274444(#2)) or Negative Control siRNA (Thermo Fisher Scientific; Stealth RNAi Negative Control Medium GC Duplexes #2) in 100 µL OPTI-MEM, then the mixture was transferred into 2 mm gap cuvettes. Cells were electroporated at 200 V for 2.5 ms twice using a NEPA21 electroporator (Nepa Gene, Ichikawa, Japan). After dilution with 10 mL culture medium, the cells were transferred into a 10 cm dish and incubated for 48 h prior to further treatment.

### 4.6. Cell Cycle Analysis by Staining with Propidium Iodide

Asynchronous, mitotic-arrested, and released tsFT210 cells were fixed with 70% EtOH in staining buffer (3% fetal bovine serum, 0.1% sodium azide in PBS (−)) for at least 4 h at −20°C. Fixed cells were washed three times with staining buffer and then labeled with staining buffer containing 50 µg/mL propidium iodide (Wako, Osaka, Japan) in the dark. Analysis of DNA content was performed by measuring the intensity of the fluorescence produced by propidium iodide using the FACSCalibur instrument (Beckton Dickinson, Bedford, MA, USA).

### 4.7. Cell Cycle Analysis by Staining with Propidium Iodide and Phospho-H3 Antibody

Asynchronous, mitotic-arrested, and released tsFT210 cells were fixed with 70% EtOH in staining buffer for at least 4 h at −20 °C. Fixed cells were washed two times with staining buffer and then permeabilized by 0.33% Triton X-100 in PBS (−). Permeabilized were washed one time with staining buffer, followed by incubation in staining buffer with phospho-H3 antibody (CST, #3377 [1:1600]) for 2 h and then with secondary antibody (coupled to AlexaFluor 488) for 30 min. Subsequently, the cells were resuspended in staining buffer containing 50 µg/mL propidium iodide and 10 µg/mL RNaseA, then incubated for 30 min at 37 °C in the dark. These cells were measured using an SH800 cell sorter (Sony, Tokyo, Japan).

### 4.8. Chromatin Immunoprecipitation (ChIP) Assay

For the ChIP assay to detect GABPA binding, asynchronous or mitotic-arrested tsFT210 cells were cross-linked in 1% formaldehyde at room temperature for 10 min and quenched with 150 mM glycine for 5 min at room temperature. Cells were washed twice in PBS and resuspended in 200 µL SDS lysis buffer (50 mM Tris pH 8,1 10 mM ethylenediaminetetraacetic acid (EDTA) pH 8.0, 1% SDS, 1 mM phenylmethyl sulfonyl fluoride (PMSF), complete in EASYPack protease inhibitor cocktail) per 5.0 × 10^6^ cells and then incubated for 10 min at 4 °C. Resuspended cells were sonicated in a bioruptor device (UCD-250; CosmoBio, Tokyo, Japan) (80 cycles 30 s on/off, high setting) and spun down for 10 min at 8 °C at maximum speed. Supernatants were diluted 10 times with ChIP dilution buffer (16.7 mM Tris pH 8.1, 1.2 mM EDTA pH 8.0, 167 mM NaCl, 1.1% Triton X-100, 0.01% SDS, Complete in EASYPack). Then, to avoid contamination of nonspecific binding substances, Dynabeads protein G (Thermo Fisher Scientific) were added and incubated for 1 h with rotation at 4 °C. Beads were immobilized on a magnet and supernatants were collected. Collected supernatants were incubated with the antibody against GABPA (Santa Cruz Biotechnology, sc-22810, [1:20]) or normal rabbit IgG (CST, #2729 [1:100]) overnight with rotation at 4 °C. The next, day, Dynabeads protein G were added and incubated for 1 h with rotation at 4 °C. Then, the beads were immobilized on a magnet and washed three times in low-salt buffer (0.1% SDS,1% Triton X-100, 2 mM EDTA, 150 mM NaCl, 20 mM Tris pH8.1), twice in high-salt buffer (0.1% SDS, 1% Triton X-100, 2 mM EDTA, 500 mM NaCl, 20 mM Tris pH 8.1), once in LiCl buffer (250 mM LiCl, 1% NP40, 1% deoxycholic acid (sodium salt), 1 mM EDTA, 10 mM Tris pH 8.1), and once in TE buffer. DNA was then eluted from the beads by incubating with 200 µL ChIP elution buffer (0.5% SDS, 5 mM EDTA, 300 mM NaCl, 10 mM Tris pH 8.1) for 6 h at 65 °C. Supernatants were treated with RNase A for 30 min and proteinase K for 1 h. After the treatment, DNA was purified using the Wizard SV Gel and PCR Clean-Up System (Promega, Madison, WI, USA).

For ChIP assay to detect histone H3, H3K9/14AC, H3K27AC, H4K5AC, SP1, Pol II, and BRD4 binding, asynchronous, mitotic-arrested, or released tsFT210 cells were cross-linked in 1% formaldehyde at room temperature for 10 min and quenched with glycine solution (10×) for 5 min at room temperature. Subsequent assays were performed using the SimpleChIP^®^ Enzymatic Chromatin IP Kit (Magnetic Beads) following manufacturer’s protocol. Antibodies used: anti-SP1 (Santa Cruz Biotechnology, sc-17824, [1:50]), anti-histone H3 (CST, #4620 [1:50]), anti-H3K9/14AC (CST, #9677 [1:50]), anti-H3K27AC (Abcam, ab4729 [1:250]), anti-H4K5AC (Abcam, EP1000Y [1:250]), anti-Pol II (Santa Cruz Biotechnology, sc-47701 [1:500]), and anti-BRD4 (Bethyl Laboratories, Montgomery, TX, USA; A301-985A50 [1:250]).

### 4.9. ChIP-Quantitative PCR and Quantitative RT-PCR

ChIP-quantitative PCR and RT-PCR were performed using the KOD SYBR qPCR Mix (Toyobo, Osaka, Japan). The sequences of each primer are described in Supplementary Tables S1 and S2. For ChIP-quantitative PCR, each ChIP sample was diluted twice and 1 µL was used in each 25 µL reaction. For quantitative RT-PCR, total RNA from cells transfected with siRNA was isolated using the FastGene RNA premium Kit (Nippon Genetics, Bunkyo-ku, Japan) and cDNA was prepared from 2.5 µg total RNA using ReverTra Ace (Toyobo) with oligo dT primer. Each cDNA was diluted twice and 1 µL was used in each 25 µL reaction. Each cDNA and ChIP sample was analyzed in duplicate. Quantitative PCR was performed using the ABI prism 7900HT (Applied Biosystems, Troy, NY, USA). The PCR cycling program was set as follows: 98 °C for 2 min followed by 40 cycles of 98 °C for 10 s and 64.4 °C for 12 s. Ct values were determined using SDS 2.3.

## Supplementary Materials

Supplementary materials can be found at https://www.mdpi.com/1422-0067/20/5/1093/s1.

## References

[1] Egli D., Birkhoff G., Eggan K. (2008). Mediators of reprogramming: Transcription factors and transitions through mitosis. Nat. Rev. Mol. Cell Biol..

[2] Martínez-Balbás M.A., Dey A., Rabindran S.K., Ozato K., Wu C. (1995). Displacement of sequence-specific transcription factors from mitotic chromatin. Cell.

[3] Prasanth K.V., Sacco-Bubulya P.A., Prasanth S.G., Spector D.L. (2003). Sequential entry of components of the gene expression machinery into daughter nuclei. Mol. Biol. Cell.

[4] Kadauke S., Blobel G.A. (2013). Mitotic bookmarking by transcription factors. Epigenetics Chromatin.

[5] Xing H., Vanderford N.L., Sarge K.D. (2008). The TBP–PP2A mitotic complex bookmarks genes by preventing condensin action. Nat. Cell Biol..

[6] Blobel G.A., Kadauke S., Wang E., Lau A.W., Zuber J., Chou M.M., Vakoc C.R. (2009). A reconfigured pattern of MLL occupancy within mitotic chromatin promotes rapid transcriptional reactivation following mitotic exit. Mol. Cell.

[7] Dey A., Nishiyama A., Karpova T., McNally J., Ozato K. (2009). Brd4 Marks Select Genes on Mitotic Chromatin and Directs Postmitotic Transcription. Mol. Biol. Cell.

[8] Zhao R., Nakamura T., Fu Y., Lazar Z., Spector D.L. (2011). Gene bookmarking accelerates the kinetics of post-mitotic transcriptional re-activation. Nat. Cell Biol..

[9] Arora M., Zhang J., Heine G.F., Ozer G., Liu H.W., Huang K., Parvin J.D. (2012). Promoters active in interphase are bookmarked during mitosis by ubiquitination. Nucleic Acids Res..

[10] Kadauke S., Udugama M.I., Pawlicki J.M., Achtman J.C., Jain D.P., Cheng Y., Hardison R.C., Blobel G.A. (2012). Tissue-specific mitotic bookmarking by hematopoietic transcription factor GATA1. Cell.

[11] Arampatzi P., Gialitakis M., Makatounakis T., Papamatheakis J. (2013). Gene-specific factors determine mitotic expression and bookmarking via alternate regulatory elements. Nucleic Acids Res..

[12] Caravaca J.M., Donahue G., Becker J.S., He X., Vinson C., Zaret K.S. (2013). Bookmarking by specific and nonspecific binding of FoxA1 pioneer factor to mitotic chromosomes. Genes Dev..

[13] Lake R.J., Tsai P.F., Choi I., Won K.J., Fan H.Y. (2014). RBPJ, the major transcriptional effector of notch signaling, remains associated with chromatin throughout mitosis, suggesting a role in mitotic bookmarking. PLoS Genet..

[14] Lodhi N., Kossenkov A.V., Tulin A.V. (2014). Bookmarking promoters in mitotic chromatin: Poly(ADP-ribose)polymerase-1 as an epigenetic mark. Nucleic Acids Res..

[15] Wong M.M., Byun J.S., Sacta M., Jin Q., Baek S.J., Gardner K. (2014). Promoter-bound p300 complexes facilitate post-mitotic transmission of transcriptional memory. PLoS ONE.

[16] Arora M., Packard C.Z., Banerjee T., Parvin J.D. (2015). RING1A and BMI1 bookmark active genes via ubiquitination of chromatin-associated proteins. Nucleic Acids Res..

[17] Lerner J., Bagattin A., Verdeguer F., Makinistoglu M.P., Garbay S., Felix T., Heidet L., Pontoglio M. (2016). Human mutations affect the epigenetic/bookmarking function of HNF1B. Nucleic Acids Res..

[18] Deluz C., Friman E.T., Strebinger D., Benke A., Raccaud M., Callegari A., Leleu M., Manley S., Suter D.M. (2016). A role for mitotic bookmarking of SOX2 in pluripotency and differentiation. Genes Dev..

[19] Festuccia N., Dubois A., Vandormael-Pournin S., Gallego Tejeda E., Mouren A., Bessonnard S., Mueller F., Proux C., Cohen-Tannoudji M., Navarro P. (2016). Mitotic binding of Esrrb marks key regulatory regions of the pluripotency network. Nat. Cell Biol..

[20] Liu Y., Pelham-Webb B., Di Giammartino D.C., Li J., Kim D., Kita K., Saiz N., Garg V., Doane A., Giannakakou P. (2017). Widespread mitotic bookmarking by histone marks and transcription factors in pluripotent stem cells. Cell Rep..

[21] Palozola K.C., Donahue G., Liu H., Grant G.R., Becker J.S., Cote A., Yu H., Raj A., Zaret K.S. (2017). Mitotic transcription and waves of gene reactivation during mitotic exit. Science.

[22] Hsiung C.C.S., Bartman C.R., Huang P., Ginart P., Stonestrom A.J., Keller C.A., Face C., Jahn K.S., Evans P., Sankaranarayanan L. (2016). A hyperactive transcriptional state marks genome reactivation at the mitosis-G1 transition. Genes Dev..

[23] Fukuoka M., Uehara A., Niki K., Goto S., Kato D., Utsugi T., Ohtsu M., Murakami Y. (2013). Identification of preferentially reactivated genes during early G1 phase using nascent mRNA as an index of transcriptional activity. Biochem. Biophys. Res. Commun..

[24] Ristevski S., O’Leary D.A., Thornell A.P., Owen M.J., Kola I., Hertzog P.J. (2004). The ETS transcription factor GABPa is essential for early embryogenesis. Mol. Cell. Biol..

[25] Manukjan G., Ripperger T., Venturini L., Stadler M., Göhring G., Schambach A., Schlegelberger B., Steinemann D. (2016). GABP is necessary for stem/progenitor cell maintenance and myeloid differentiation in human hematopoiesis and chronic myeloid leukemia. Stem Cell Res..

[26] Yu S., Cui K., Jothi R., Zhao D.M., Jing X., Zhao K., Xue H.H. (2011). GABP controls a critical transcription regulatory module that is essential for maintenance and differentiation of hematopoietic stem/progenitor cells. Blood.

[27] Yang Z.F., Mott S., Rosmarin A.G. (2007). The Ets transcription factor GABP is required for cell-cycle progression. Nat. Cell Biol..

[28] Yang Z.F., Drumea K., Mott S., Wang J., Rosmarin A.G. (2014). GABP transcription factor (nuclear respiratory factor 2) is required for mitochondrial biogenesis. Mol. Cell. Biol..

[29] O’Connor L., Gilmour J., Bonifer C. (2016). The role of the ubiquitously expressed transcription factor Sp1 in tissue-specific transcriptional regulation and in disease. Yale J. Biol. Med..

[30] Chuang J.Y., Wang S.A., Yang W.B., Yang H.C., Hung C.Y., Su T.P., Chang W.C., Hung J.J. (2012). Sp1 phosphorylation by cyclin-dependent kinase 1/cyclin B1 represses its DNA-binding activity during mitosis in cancer cells. Oncogene.

[31] Segil N., Roberts S.B., Heintz N. (1991). Mitotic phosphorylation of the Oct-1 homeodomain and regulation of Oct-1 DNA binding activity. Science.

[32] Roberts S.B., Segil N., Heintz N. (1991). Differential phosphorylation of the transcription factor Octl during the cell cycle. Science.

[33] Dovat S., Ronni T., Russell D., Ferrini R., Cobb B.S., Smale S.T. (2002). A common mechanism for mitotic inactivation of C2H2 zinc finger DNA-binding domains. Genes Dev..

[34] Rizkallah R., Alexander K.E., Hurt M.M. (2011). Global mitotic phosphorylation of C2H2zinc finger protein linker peptides. Cell Cycle.

[35] Róna G., Borsos M., Ellis J.J., Mehdi A.M., Christie M., Környei Z., Neubrandt M., Tóth J., Bozóky Z., Buday L. (2014). Dynamics of re-constitution of the human nuclear proteome after cell division is regulated by NLS-adjacent phosphorylation. Cell Cycle.

[36] Rizkallah R., Batsomboon P., Dudley G.B., Hurt M.M. (2015). Identification of the oncogenic kinase TOPK/PBK as a master mitotic regulator of C2H2 zinc finger proteins. Oncotarget.

[37] Sekiya T., Murano K., Kato K., Kawaguchi A., Nagata K. (2017). Mitotic phosphorylation of CCCTC-binding factor (CTCF) reduces its DNA binding activity. FEBS Open Bio.

[38] Raccaud M., Suter D.M. (2017). Transcription factor retention on mitotic chromosomes: Regulatory mechanisms and impact on cell fate decisions. FEBS Lett..

[39] Galvagni F., Capo S., Oliviero S. (2001). Sp1 and Sp3 physically interact and co-operate with GABP for the activation of the utrophin promoter. J. Mol. Biol..

[40] Rudge T.L., Johnson L.F. (2002). Synergistic activation of the TATA-less mouse thymidylate synthase promoter by the Ets transcription factor GABP and Sp1. Exp. Cell Res..

[41] Kruhlak M.J., Hendzel M.J., Fischle W., Bertos N.R., Hameed S., Yang X.J., Verdin E., Bazett-Jones D.P. (2001). Regulation of global acetylation in mitosis through loss of histone acetyltransferases and deacetylases from chromatin. J. Biol. Chem..

[42] Valis E., Sánchez-Molina S., Martínez-Balbás M.A. (2005). Role of histone modifications in marking and activating genes through mitosis. J. Biol. Chem..

[43] Young D.W., Hassan M.Q., Pratap J., Galindo M., Zaidi S.K., Lee S., Yang X., Xie R., Javed A., Underwood J.M. (2007). Mitotic occupancy and lineage-specific transcriptional control of rRNA genes by Runx2. Nature.

[44] Young D.W., Hassan M.Q., Yang X.Q., Galindo M., Javed A., Zaidi S.K., Furcinitti P., Lapointe D., Montecino M., Lian J.B. (2007). Mitotic retention of gene expression patterns by the cell fate-determining transcription factor Runx2. Proc. Natl. Acad. Sci. USA.

[45] Ali S.A., Zaidi S.K., Dobson J.R., Shakoori A.R., Lian J.B., Stein J.L., van Wijnen A.J., Stein G.S. (2010). Transcriptional corepressor TLE1 functions with Runx2 in epigenetic repression of ribosomal RNA genes. Proc. Natl. Acad. Sci. USA.

[46] Festuccia N., Owens N., Papadopoulou T., Gonzalez I., Tachtsidi A., Vandoermel-Pournin S., Gallego E., Gutierrez N., Dubois A., Cohen-Tannoudji M. (2019). Transcription factor activity and nucleosome organisation in mitosis. Genome Res..

[47] Akıncılar S.C., Khattar E., Boon P.L., Unal B., Fullwood M.J., Tergaonkar V. (2016). Long-range chromatin interactions drive mutant TERT promoter activation. Cancer Discov..

[48] Cao R., Wang L., Wang H., Xia L., Erdjument-Bromage H., Tempst P., Jones R.S., Zhang Y. (2002). Role of histone H3 lysine 27 methylation in polycomb-group silencing. Science.

[49] Tie F., Banerjee R., Saiakhova A.R., Howard B., Monteith K.E., Scacheri P.C., Cosgrove M.S., Harte P.J. (2014). Trithorax monomethylates histone H3K4 and interacts directly with CBP to promote H3K27 acetylation and antagonize Polycomb silencing. Development.

[50] Schmitges F.W., Prusty A.B., Faty M., Stützer A., Lingaraju G.M., Aiwazian J., Sack R., Hess D., Li L., Zhou S. (2011). Histone methylation by PRC2 is inhibited by active chromatin marks. Mol. Cell.

[51] Steffen P.A., Fonseca J.P., Gänger C., Dworschak E., Kockmann T., Beisel C., Ringrose L. (2013). Quantitative in vivo analysis of chromatin binding of Polycomb and Trithorax group proteins reveals retention of ASH1 on mitotic chromatin. Nucleic Acids Res..

[52] Zhang Y., Tuzova M., Xiao Z.X., Cruikshank W.W., Center D.M. (2008). Pro-IL-16 recruits histone deacetylase 3 to the Skp2 core promoter through interaction with transcription factor GABP. J. Immunol..

[53] Ravel-Chapuis A., Vandromme M., Thomas J.L., Schaeffer L. (2007). Postsynaptic chromatin is under neural control at the neuromuscular junction. EMBO J..

