# Supplementary material for: Identification of GA-Binding Protein Transcription Factor Alpha Subunit (GABPA) as a Novel Bookmarking Factor
Source: Int J Mol Sci. 2019 Mar 4;20(5):1093. doi: 10.3390/ijms20051093 (PMC6429373; doi:10.3390/ijms20051093)
Supplement: Supplementary file 1 [file ijms-20-01093-s001.pdf]

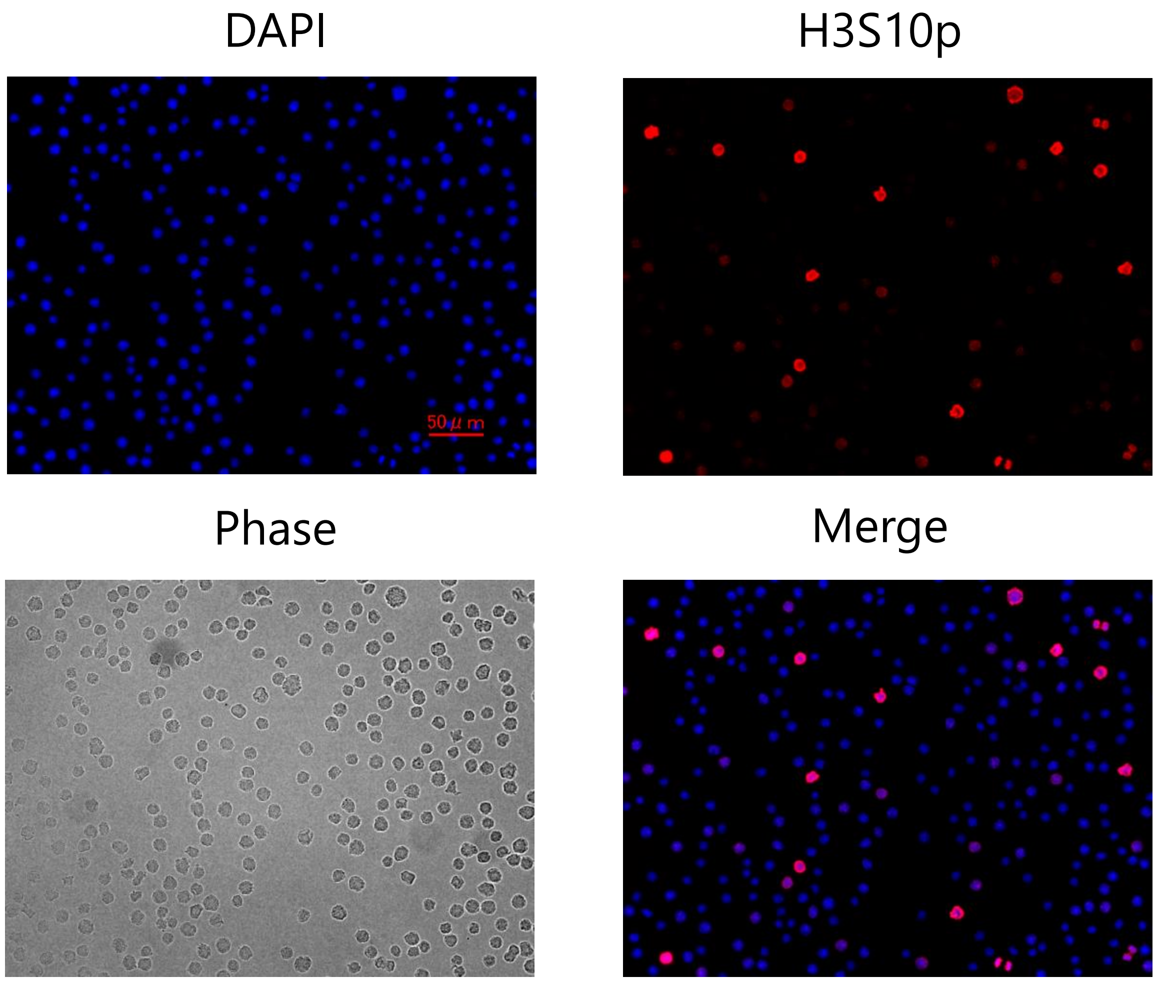

Average percentage of mitotic cells : 12.40 ± 1.22%

Supplementary Figure S1. Asynchronous tsFT210 cells stained with phosphorylation at Ser10 of histone H3. The cells were immunostained with anti- phosphorylation at Ser10 of histone H3 (red, upper right) antibodies and the DNA was counterstained with DAPI (blue, upper left).

The percentage of mitotic cells vs total cells was measured from a minimum of 100 cells counted from each slide. The average of those percentage and standard deviation calculated from 5 experiments was shown below the figure.

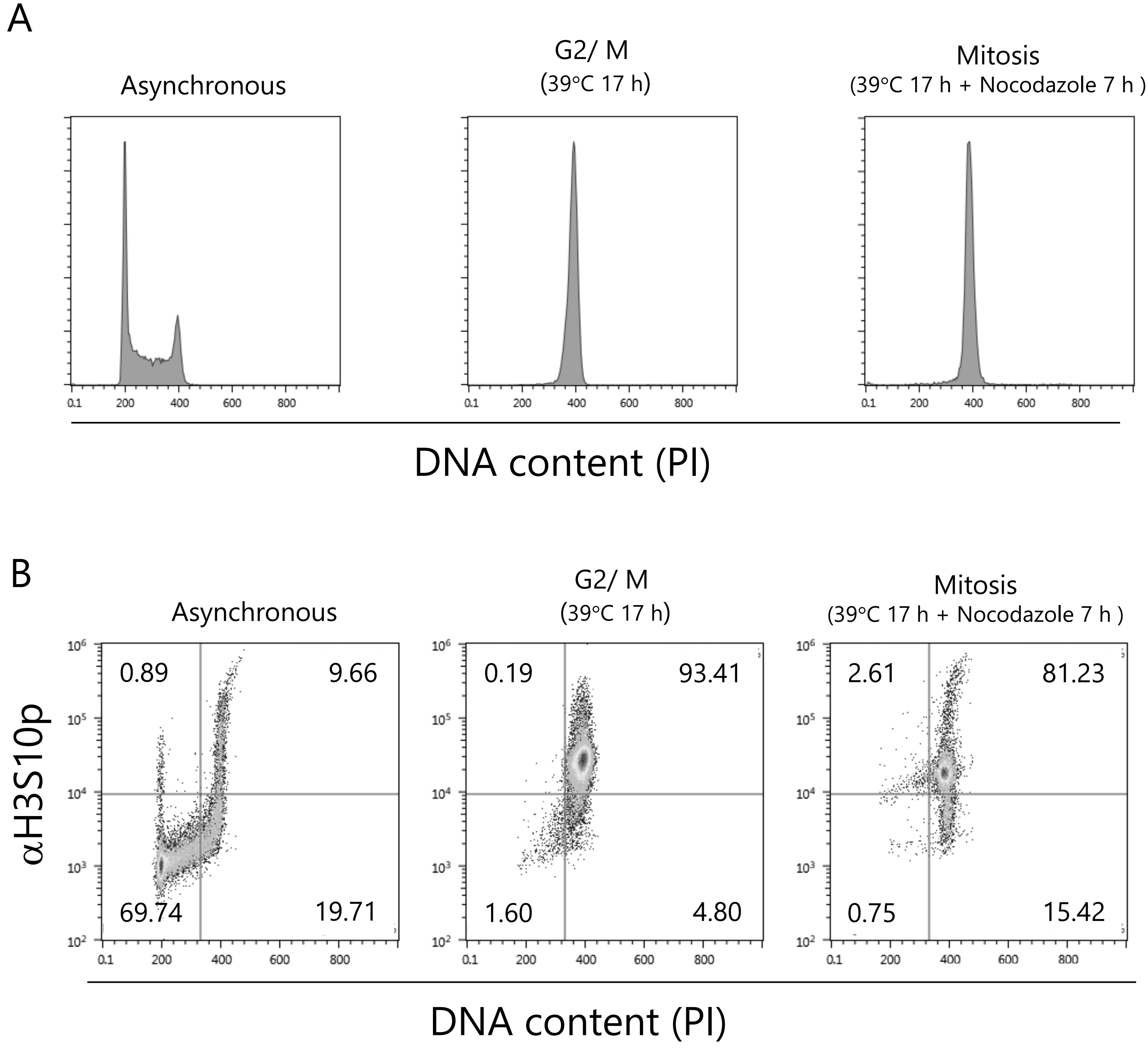

Supplementary Figure S2. Cell cycle synchronization at the mitosis (A) Asynchronous, G2/M- arrested and mitotic- arrested tsFT210 cells were harvested and stained with propidium iodide (PI). DNA content of these cells were analyzed by FACS. (B) Asynchronous, G2/M- arrested and mitotic- arrested tsFT210 cells were harvested and stained with propidium iodide (PI) and an antibody against phosphorylation at Ser10 of histone H3 (aH3S10p). DNA content and H3S10p were analyzed by FACS.

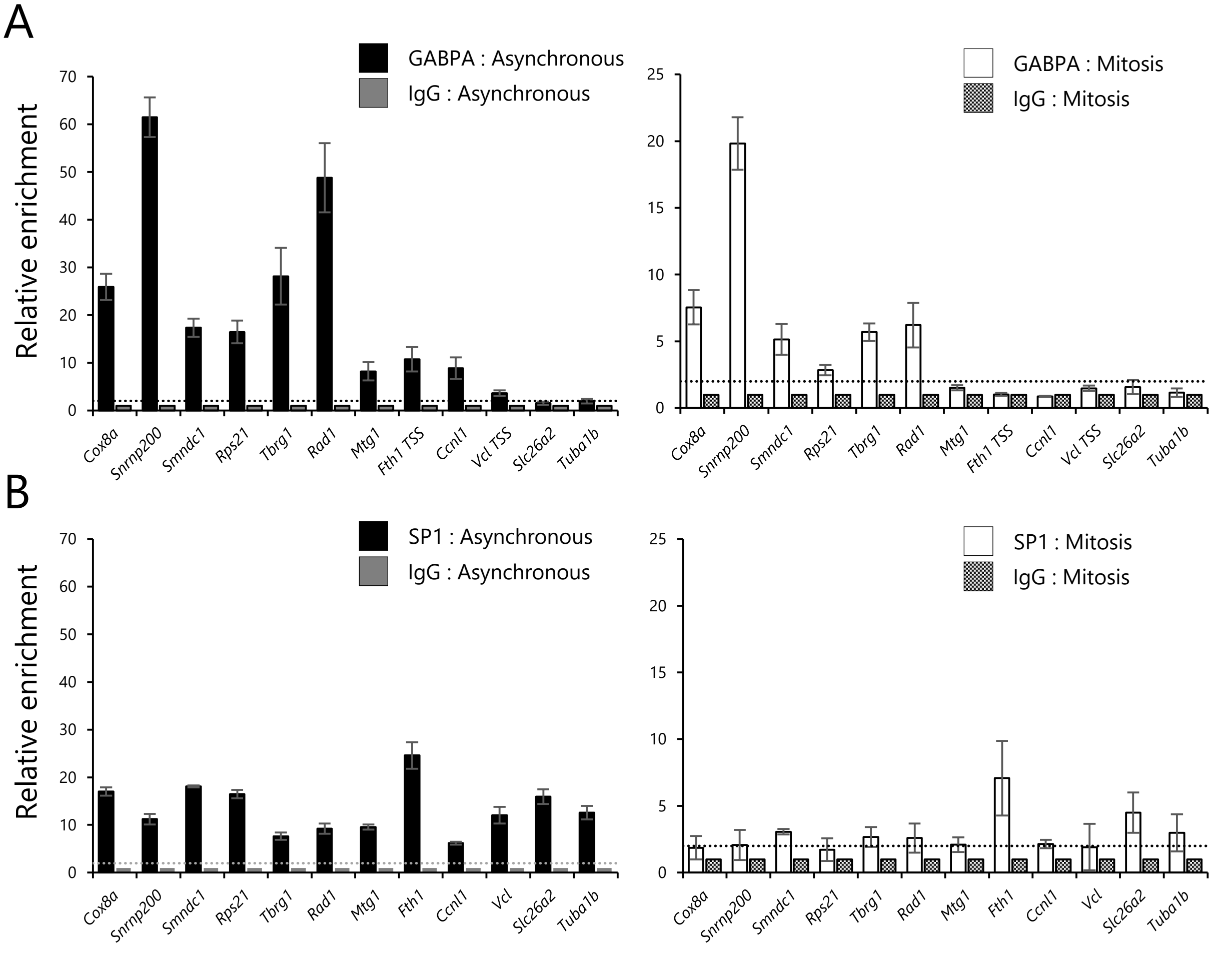

Supplementary Figure S3. Relative enrichment of the GA-binding protein transcription factor alpha subunit (GABPA) (A) and Sp1 transcription factor (SP1) (B) binding versus IgG in asynchronous cells (left side) and mitotic-arrested cells (right side) as shown in Fig. 1B, C. Dotted lines represent the two-fold values of normal IgG binding which preset . Error bars denote SEM (n = 3).

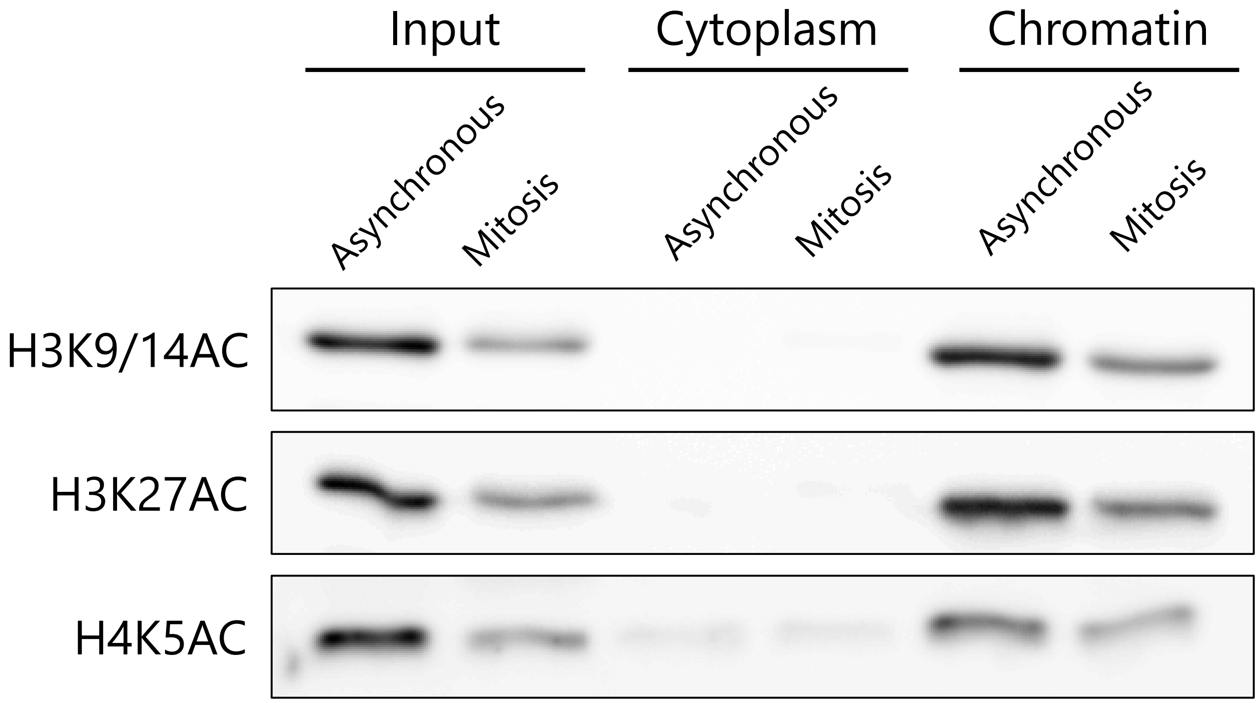

Supplementary Figure S4. Protein levels of H3K9/14AC, H3K27AC and H4K5AC in cytoplasmic and chromatin fractions. Asynchronous and mitosis-arrested tsFT210 cells were fractionated into cytoplasmic and chromatin fractions. . The fractions were then analyzed by western blot with indicated antibodies.

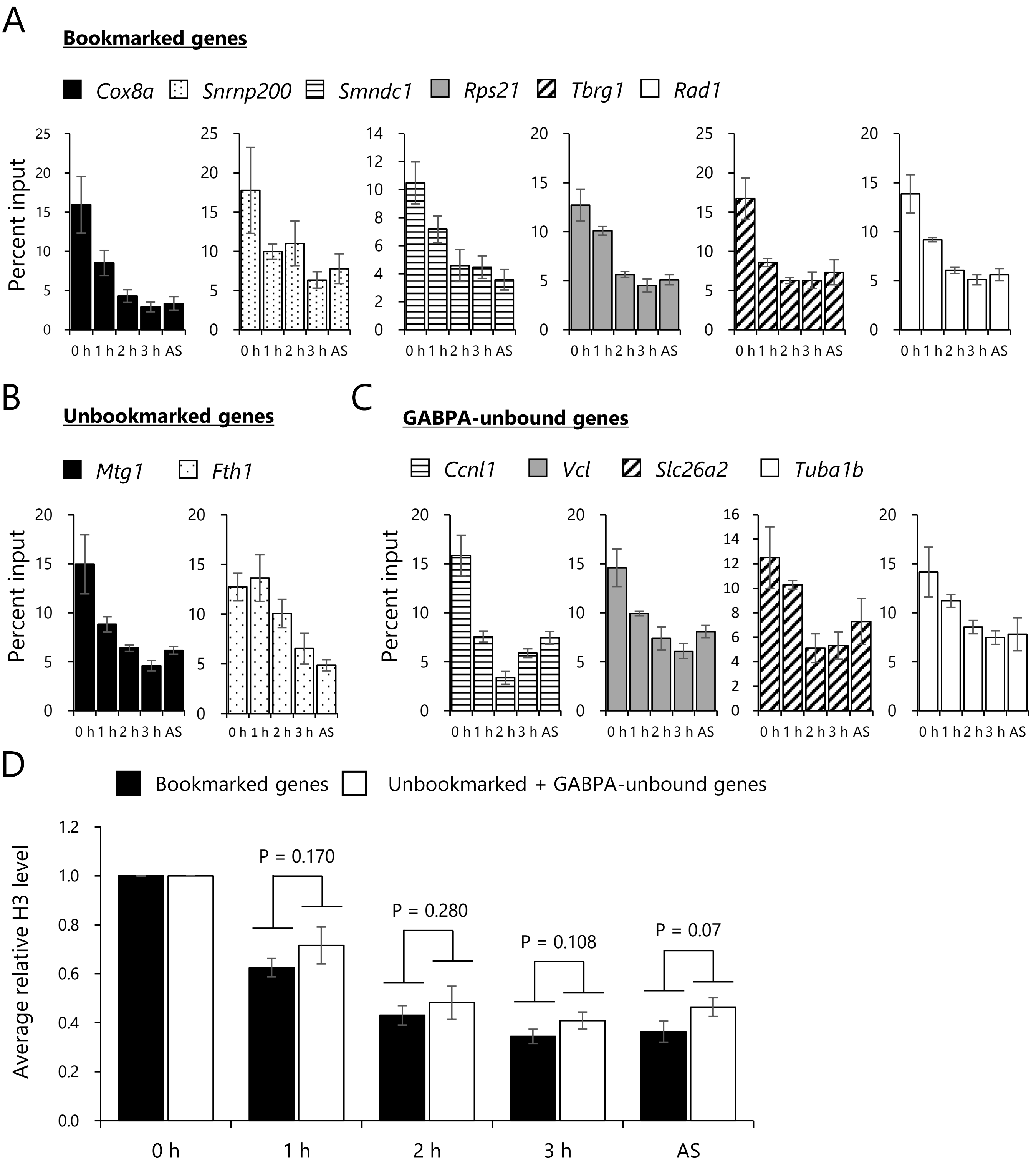

Supplementary Figure S5. Histone H3 level in cells at indicated time points after release from mitotic synchronization and asynchronous (AS) cells. (A-C) Mitotic-arrested tsFT210 cells were released and harvested at indicated time points, then subjected to chromatin immunoprecipitation assay using antibodies against histone H3, followed by quantitative PCR using primer sets specific for upstream regions of the bookmarked (A), unbookmarked (B) and GABPA-unbound (C) genes. Error bars denote SEM (n = 3). (D) Comparison of the average relative H3 level in cells at indicated time points after release from mitotic synchronization at the bookmarked vs those unbookmarked gene regions. Error bars denote SEM (n = 3). The significance of differences was analyzed by a one-tailed Student's t-test.

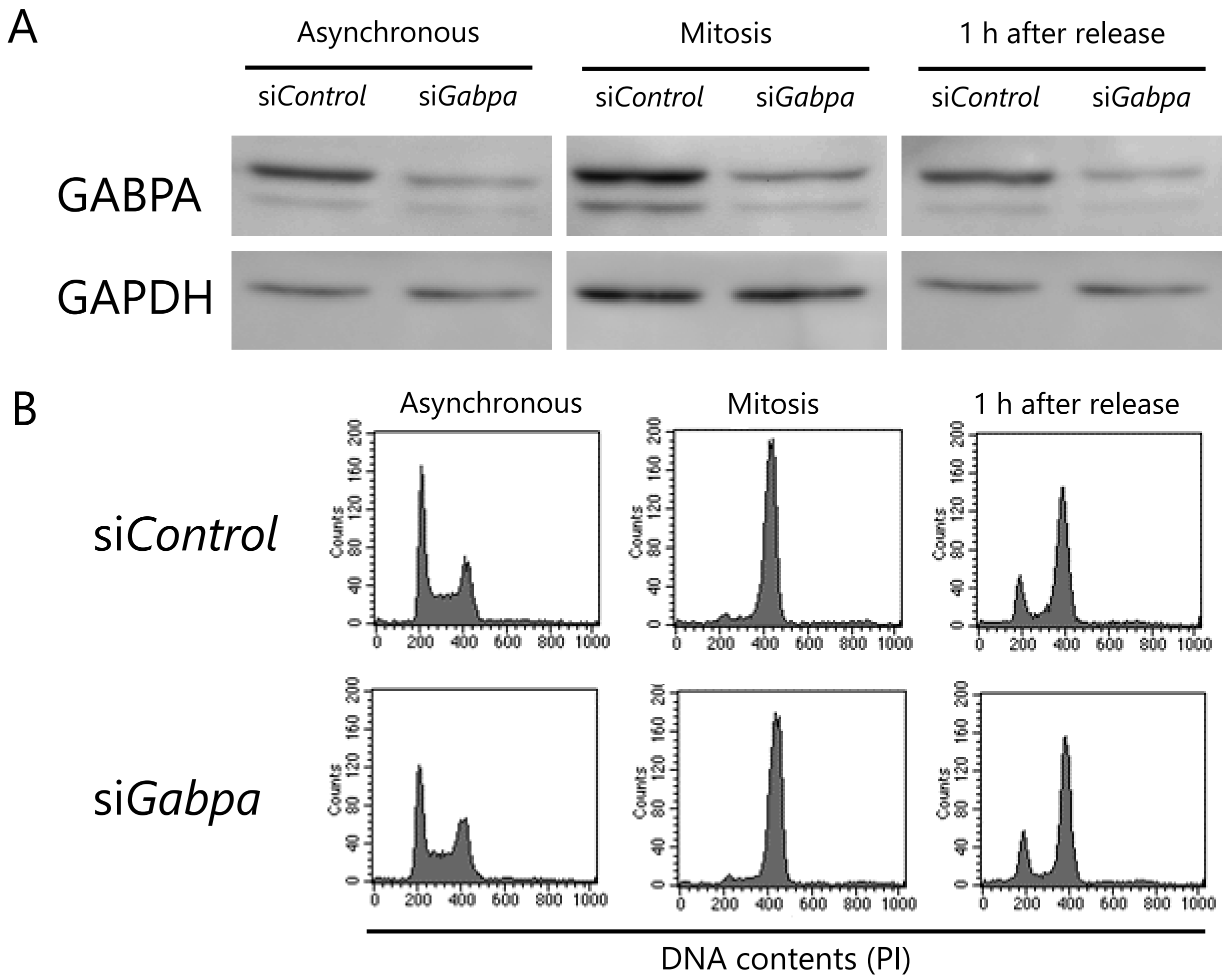

Supplementary Figure S6. Cell cycle progression after release from mitotic arrest at *Gabpa* knockdown cells. (A) Protein levels of GABPA in *Gabpa* Knockdown cells. tsFT210 cells transfected with siRNA against *Gabpa* or negative control siRNA were arrested in mitosis, then released from mitotic arrest and harvested after 1 h. The protein levels of GABPA in *Gabpa* knockdown asynchronous, mitosis-arrested and 1h after release from nocodazole arrest tsFT210 cells were analyzed by western blot with indicated antibodies. GAPDH was used as loading control.
(B) Cell cycle stage distribution analyzed by FACS. tsFT210 cells transfected with siRNA against *Gabpa* or negative control were arrested in mitosis, then released from mitotic arrest and harvested after 1 h. DNA content of these cells were analyzed by FACS.

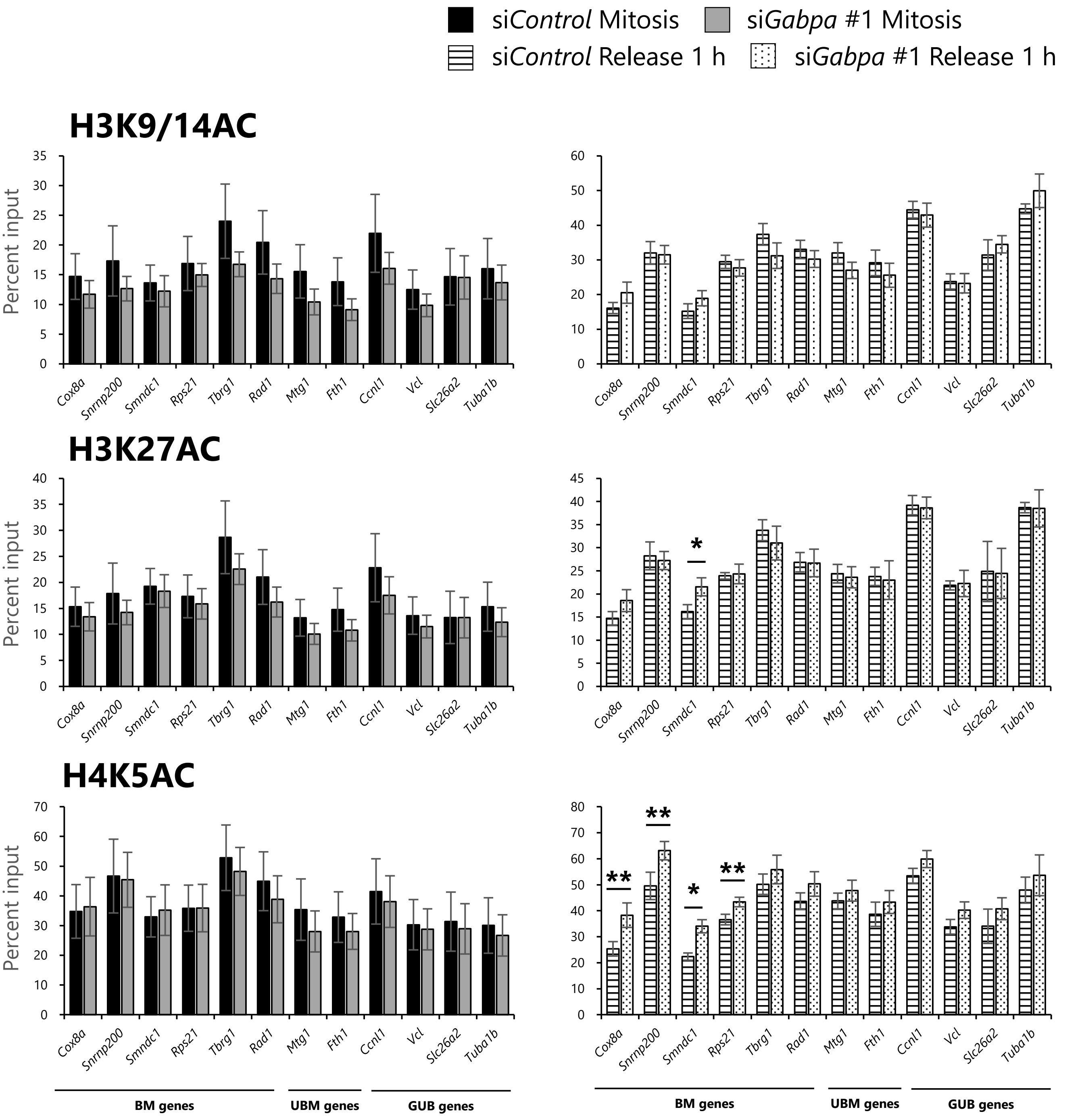

Supplementary Figure S7. Absolute percent input values of the ChIP assay described in Fig. 4. Histone H3K9/14, H3K27, and H4K5 acetylation levels in *Gabpa* knockdown or transfected with negative control siRNA mitosis-arrested and 1h after release from mitotic arrest tsFT210 cells. Mitosis-arrested and 1h after release from mitotic arrest tsFT210 were harvested, then subjected to ChIP assay using antibodies against to H3K9/14AC, H3K27AC and H4K5AC, followed by quantitative PCR assay using primer sets specific for upstream regions of the bookmarked (BM), unbookmarked (UBM) and GABPA-unbound (GUB) genes. Error bars denote SEM (n = 3) asterisks indicate significance by a one-tailed Student's t-test: (**)P < 0.05,(*)P < 0.1.

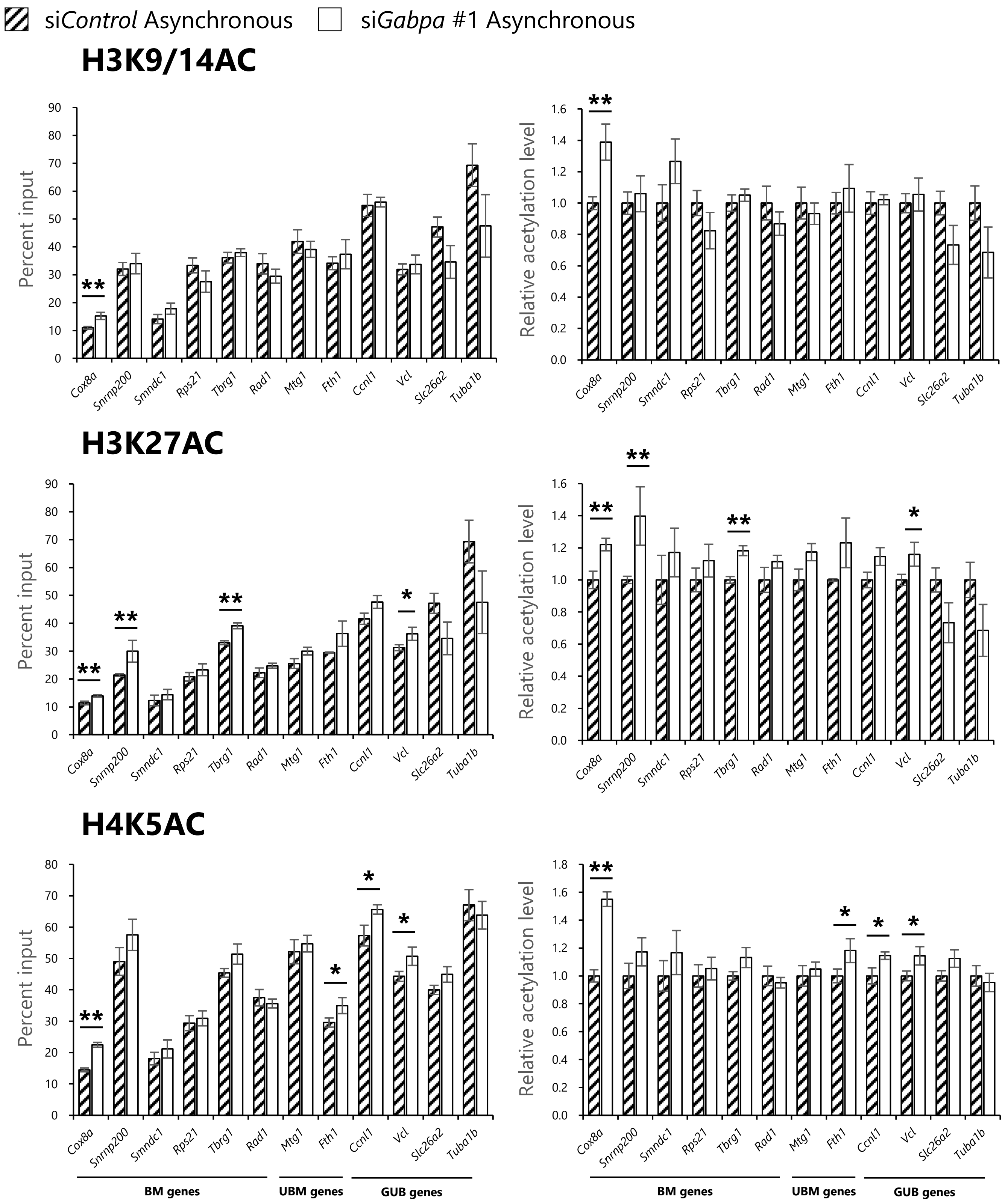

Supplementary Figure S8. *Gabpa* knockdown increases histone acetylation levels at several bookmarked, unbookmarked and GABPA-unbound genes in asynchronous cells. Asynchronous tsFT210 cells were harvested, then subjected to ChIP assay using antibodies against to H3K9/14AC, H3K27AC and H4K5AC, followed by quantitative PCR assay using primer sets specific for upstream regions of the bookmarked (BM), unbookmarked (UBM) and GABPA-unbound (GUB) genes. In each figure, the left side graph shows raw data plotted as percent input DNA and the right side graph shows the same data replotted relative to the si*Control* acetylation level. Error bars denote SEM (n = 3) asterisks indicate significance by a one-tailed Student's t-test: (**)P < 0.05, (*)P < 0.1

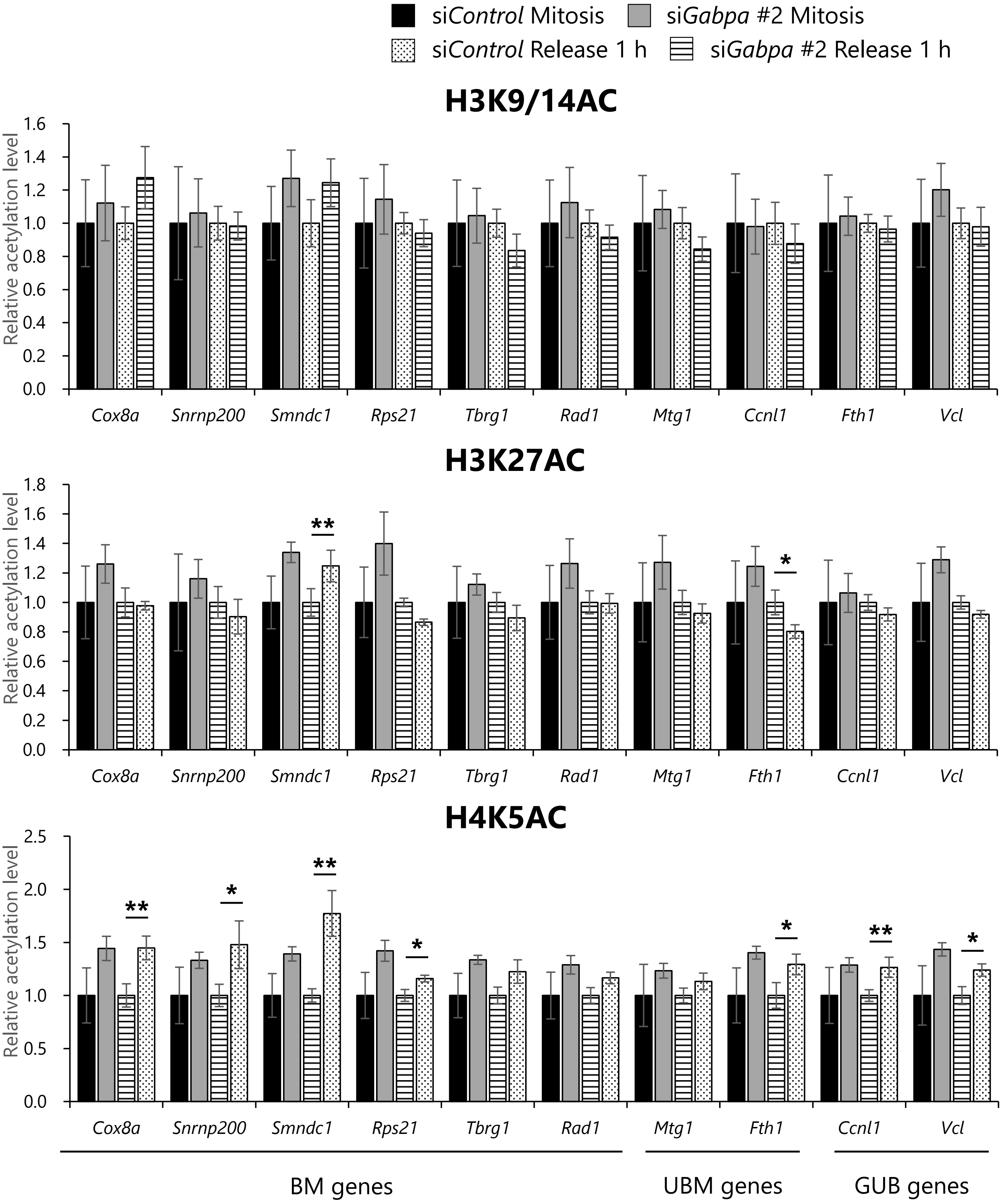

Supplementary Figure S9. The same assay described in Fig. 4 using different siRNA. Histone H3K9/14, H3K27, and H4K5 acetylation levels in *Gabpa* knockdown or transfected with negative control siRNA mitosis-arrested and 1h after release from mitotic arrest tsFT210 cells. Mitosis-arrested, 1h after release from mitotic arrest tsFT210 were harvested, then subjected to ChIP assay using antibodies against to H3K9/14AC, H3K27AC and H4K5AC, followed by quantitative PCR assay using primer sets specific for upstream regions of the bookmarked (BM), unbookmarked (UBM) and GABPA-unbound (GUB) genes. Error bars denote SEM (n = 3) asterisks indicate significance by a one-tailed Student's t-test: (**)P < 0.05, (*)P < 0.1.

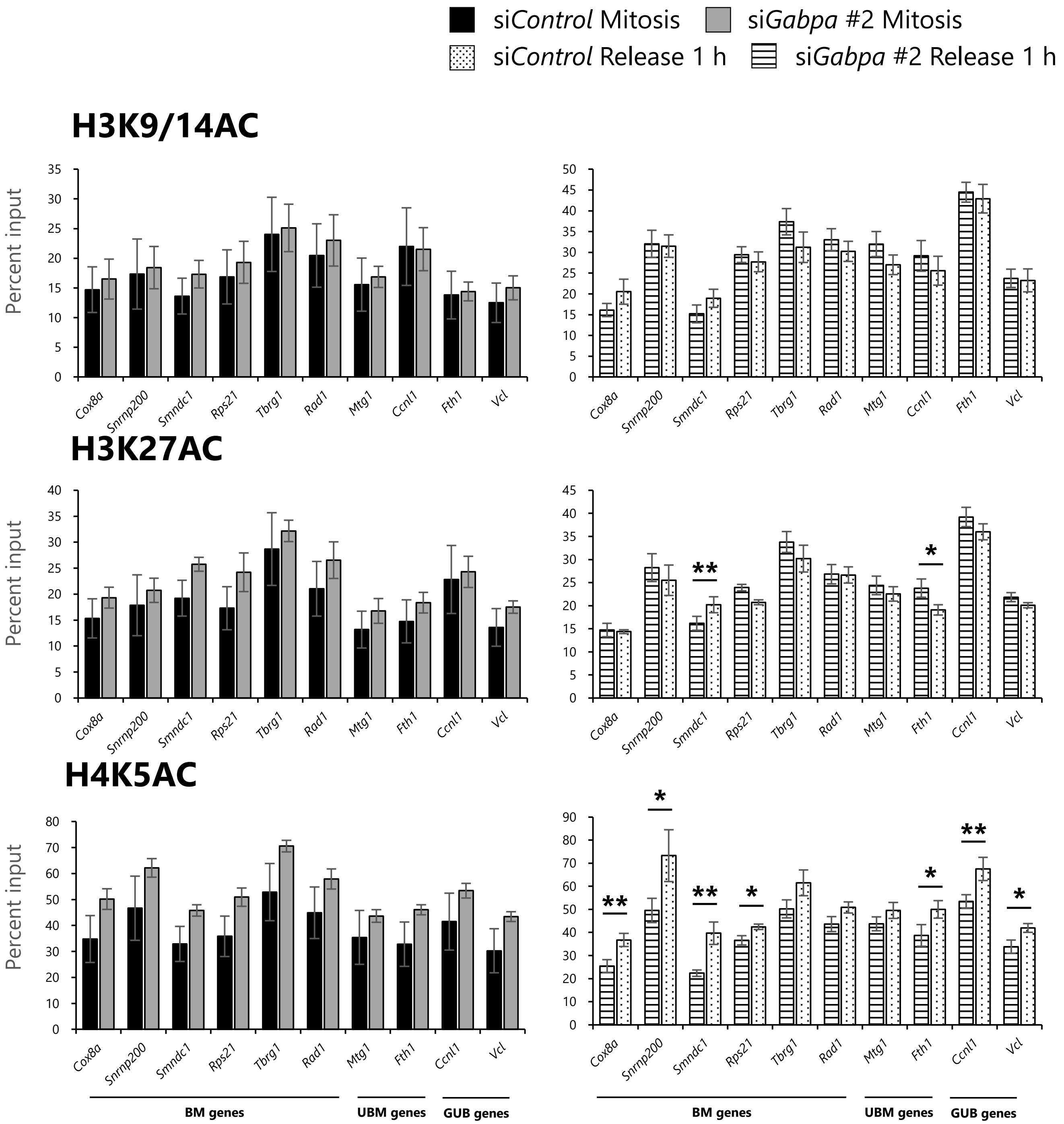

Supplementary Figure S10. Absolute percent input values of the ChIP assay described in Fig. S6 using different siRNA. Histone H3K9/14, H3K27, and H4K5 acetylation levels in *Gabpa* knockdown or transfected with negative control siRNA mitosis-arrested and 1h after release from mitotic arrest tsFT210 cells. Mitosis-arrested and 1h after release from mitotic arrest tsFT210 were harvested, then subjected to ChIP assay using antibodies against to H3K9/14AC, H3K27AC and H4K5AC, followed by quantitative PCR assay using primer sets specific for upstream regions of the bookmarked (BM), unbookmarked (UBM) and GABPA-unbound (GUB) genes. Error bars denote SEM (n = 3) asterisks indicate significance by a one-tailed Student's t-test: (**)P < 0.05,(*)P < 0.1.

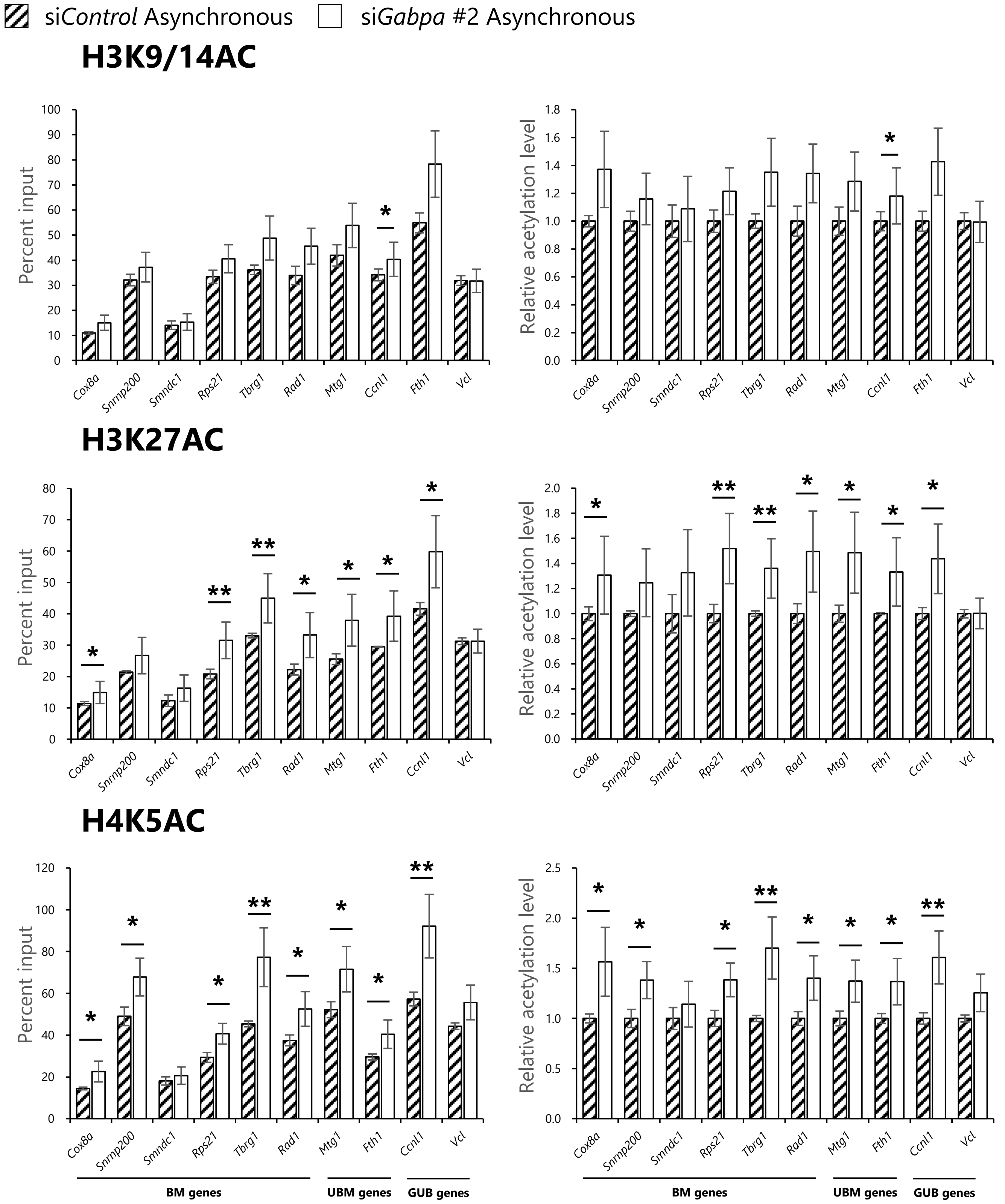

Supplementary Figure S11. The same assay described in Fig. S7 using different siRNA. Asynchronous tsFT210 cells were harvested, then subjected to ChIP assay using antibodies against to H3K9/14AC, H3K27AC and H4K5AC, followed by quantitative PCR assay using primer sets specific for upstream regions of the bookmarked (BM), unbookmarked (UBM) and GABPA-unbound (GUB) genes. In each figure, the left side graph shows raw data plotted as percent input DNA and the right side graph shows the same data replotted relative to the si*Control* acetylation level. Error bars denote SEM (n = 3) asterisks indicate significance by a one-tailed Student's t-test: (**)P < 0.05, (*)P < 0.1

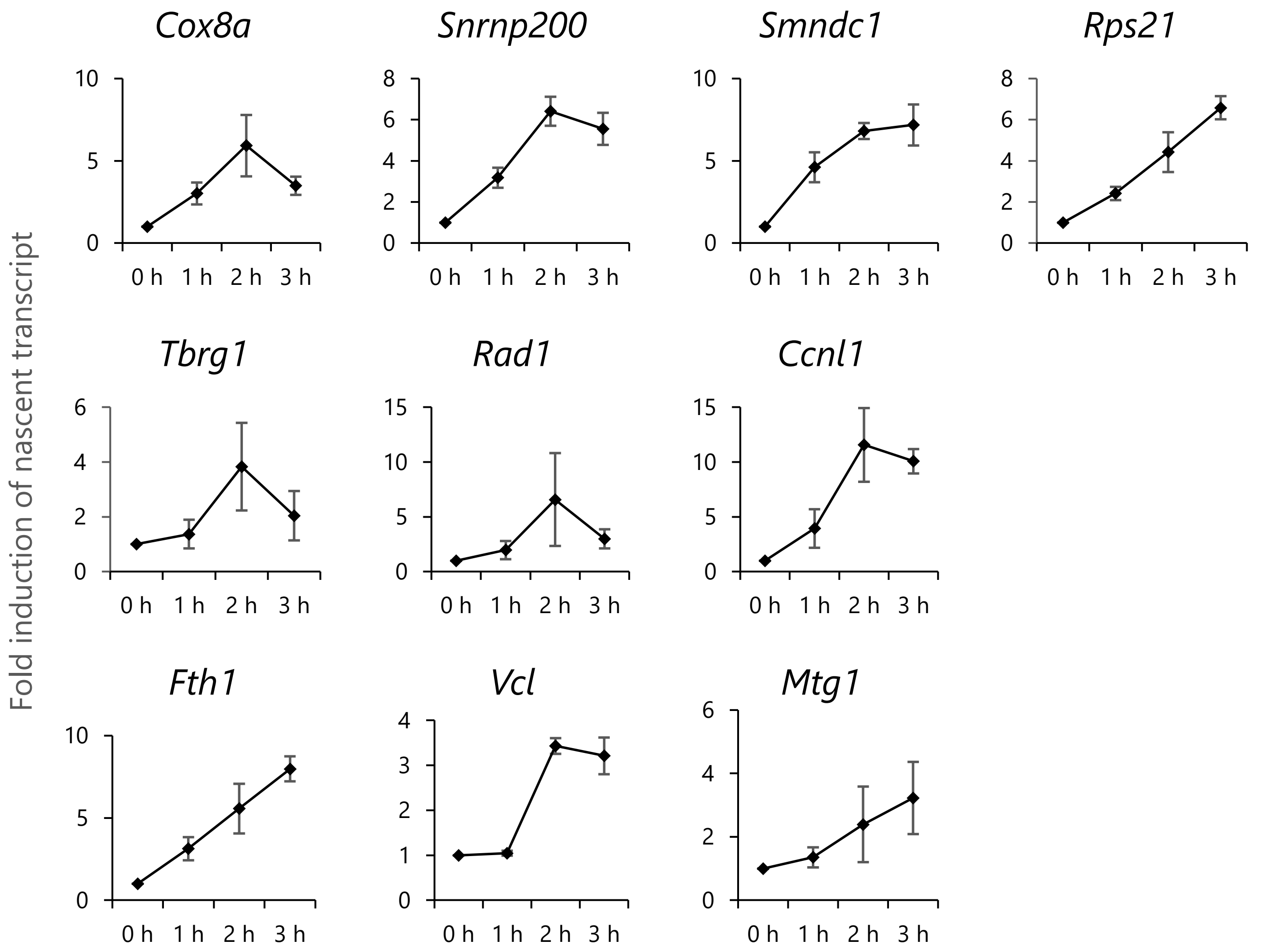

Supplementary Figure S12. Reassessment of the transcriptional reactivation during M/G1 transition by quantitative RT-PCR. Mitosis-arrested tsFT210 cells were released and harvested at indicated time points, followed by quantitative RT-PCR assay using primer sets specific for the nascent transcripts of the several genes reactivated during M/G1 transition early phase or mid phase. Data represent relative values of indicated time points versus 0 h. Error bars denote SEM (n = 3).

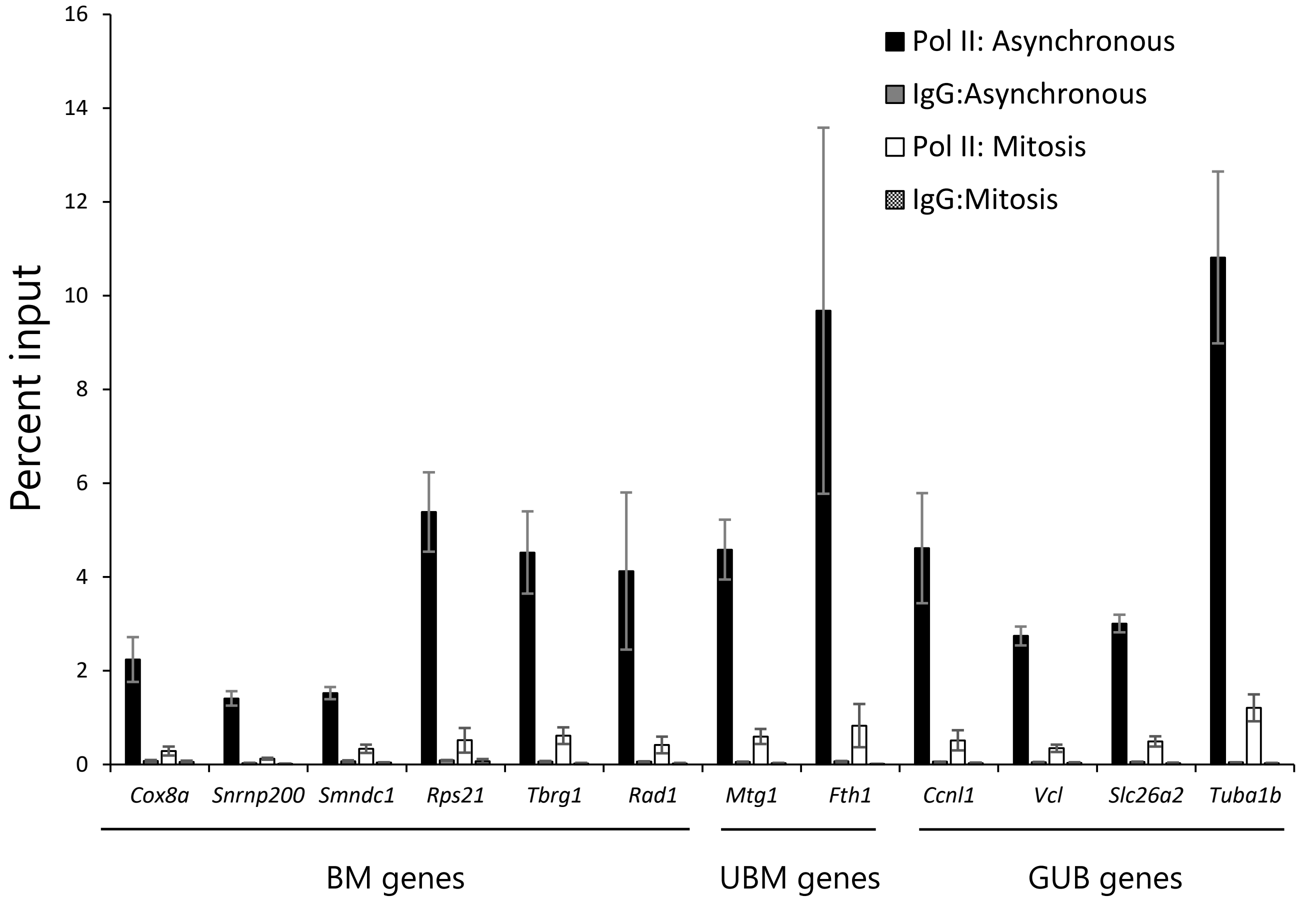

Supplementary Figure S13. The binding of Pol II at upstream regions of the genes reactivated during M/G1 transition in asynchronous and mitosis-arrested tsFT210 cells. Asynchronous and mitosis-arrested tsFT210 cells were subjected to ChIP assay using antibodies against to Pol II, followed by quantitative PCR assay using primer sets specific for the upstream of the bookmarked (BM), unbookmarked (UBM) and GABPA-unbound (GUB) genes. Normal rabbit IgG was used as a negative control. Error bars denote SEM (n = 3).

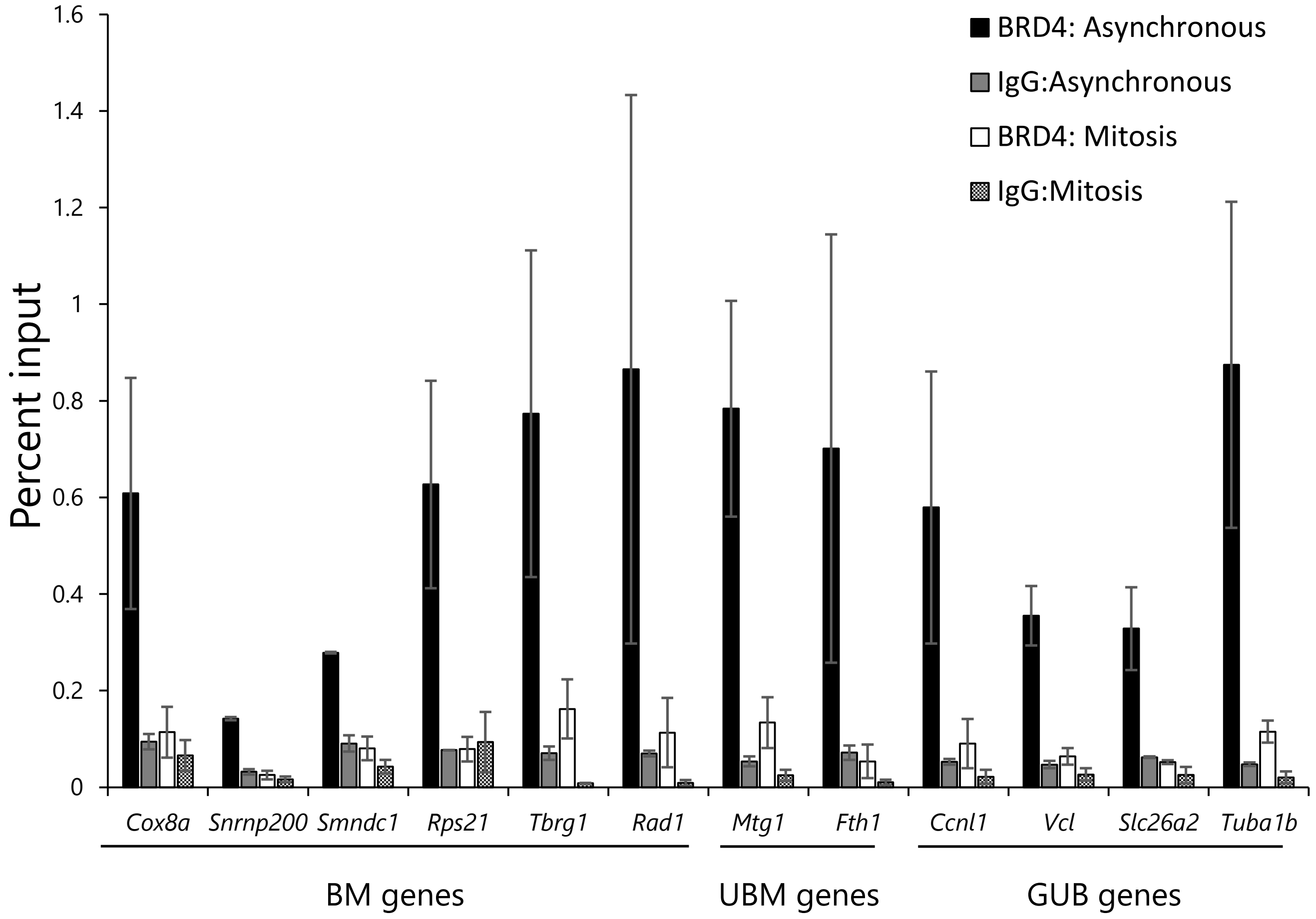

Supplementary Figure S14. The binding of BRD4 at upstream regions of the genes reactivated during M/G1 transition in asynchronous and mitosis-arrested tsFT210 cells. Asynchronous and mitosis-arrested tsFT210 cells were subjected to ChIP assay using antibodies against to BRD4, followed by quantitative PCR assay using primer sets specific for the upstream regions of the bookmarked (BM), unbookmarked (UBM) and GABPA-unbound (GUB) genes. Normal rabbit IgG was used as a negative control. Error bars denote SEM (n = 2).

Supplementary Table S1. Primer sequences for ChIP-quantitative PCR

ChIP-qPCR primers	Forward(5' - 3')	Reverse(5' - 3')
Cox8a	TGTGTAGGATGATGTCGGTTGG	GGAAATGACGTCGGGACAAG
Snrnp200	CGTTTCTTTTCTCCGTCCCTTAC	ACTCAGGGTTCCACTCTTCTTCAC
Smndc1	GCGGAACTGACGTAAAACGAAC	CGGACTCTCGTGGAAAAGATG
Rps21	TATGGTGGATACGAGTACCCTGAC	AGAGCGCAGAAAGGAAGTAGCTC
Tbrg1	GAACCACCGCAACTTCCAAAC	ACTTCCCAGTCGATTTCCCAAG
Rad1	AGTTAAGTCATCCGGCTCCAAG	ACCGTTCAGATACGCAACCAC
Mtg1	TTCCGACACCAGTGACATGAG	AACCAGCGTGCTACATCGTG
Fth1	ACACGCCCACAGGAAGAGG	CAAGCACTGTTGAAGCAGGAAAC
Ccnl1	CAGACCGTGCAGGGGTATC	CAGGGGTGGTAATGGCTTTAGTC
Vcl	GTGACGGTAAAGGGGAGCAC	TCCGCAGGATCACCTCAGTAG
Slc26a2	GGTTACTTCCGCACCTTTACTCC	CTCCCTTTAATACCCGGCAAC
Tuba1b	ATCTCTCACCCTCGCCTTCTAAC	CTTCTTCCTCCCCTCCATCAC

Supplementary Table S2. Primer sequences for pre-mRNA quantitative RT-PCR

qRT-PCR primers	Forward(5' - 3')	Reverse(5' - 3')
Cox8a	ACTTCCCATGCCTAAGGACTACC	AGGCAGAAGACAACACACGAAG
Snrnp200	GGCCATCAAGTTTTACATGCTC	CTCCAATCACAACCCACCAG
Smndc1	AGAGGACCAGAAGGTGAAATGG	AGCAACAAAGTGAGGAGGGATG
Rps21	AGCCAGCTCTGTGTGGATACTC	GACAATTCCATCAGCCTTAGCC
Tbrg1	GATGCAACTATGAGCCTTGAAGC	ACGTGTGATGAGGATACAGAAGG
Rad1	CACTGTGATAAGACCCAGGTCAAC	CCCAAGTAAATCTAGTGCCAACC
Mtg1	CCATGGCCGATTATCTCCTTTAC	ACAGCATGAGCAAGAAGGGAAC
Ccnl1	CTGTTGCCAGTAGACACTGAACC	CGAGACCTGCTTCTTGATCTTG
Fth1	TGGTAGATGACTTGACCCGATG	AGTAGCCAGTTTGTGCAGTTCC
Vcl	GGAGTTGGCTATGAACTTGTGG	TGAGCTGGGTGCTTATAGTTGG
